# Redundant Trojan horse and endothelial-circulatory mechanisms for host-mediated spread of *Candida albicans* yeast

**DOI:** 10.1371/journal.ppat.1008414

**Published:** 2020-08-10

**Authors:** Allison K. Scherer, Bailey A. Blair, Jieun Park, Brittany G. Seman, Joshua B. Kelley, Robert T. Wheeler

**Affiliations:** 1 Department of Molecular & Biomedical Sciences, University of Maine, Orono, Maine, United States of America; 2 Graduate School of Biomedical Sciences, University of Maine, Orono, Maine, United States of America; 3 Department of Cell Biology and Department of Pharmacology and Cancer Biology, Duke University, Durham, North Carolina, United States of America; University of Birmingham, UNITED KINGDOM

## Abstract

The host innate immune system has developed elegant processes for the detection and clearance of invasive fungal pathogens. These strategies may also aid in the spread of pathogens *in vivo*, although technical limitations have previously hindered our ability to view the host innate immune and endothelial cells to probe their roles in spreading disease. Here, we have leveraged zebrafish larvae as a model to view the interactions of these host processes with the fungal pathogen *Candida albicans in vivo*. We examined three potential host-mediated mechanisms of fungal spread: movement inside phagocytes in a “Trojan Horse” mechanism, inflammation-assisted spread, and endothelial barrier passage. Utilizing both chemical and genetic tools, we systematically tested the loss of neutrophils and macrophages and the loss of blood flow on yeast cell spread. Both neutrophils and macrophages respond to yeast-locked and wild type *C*. *albicans* in our model and time-lapse imaging revealed that macrophages can support yeast spread in a “Trojan Horse” mechanism. Surprisingly, loss of immune cells or inflammation does not alter dissemination dynamics. On the other hand, when blood flow is blocked, yeast can cross into blood vessels but they are limited in how far they travel. Blockade of both phagocytes and circulation reduces rates of dissemination and significantly limits the distance of fungal spread from the infection site. Together, this data suggests a redundant two-step process whereby (1) yeast cross the endothelium inside phagocytes or via direct uptake, and then (2) they utilize blood flow or phagocytes to travel to distant sites.

## Author summary

Although *Candida albicans* is the most common cause of fungal bloodstream infection, we know little about how it spreads from one tissue to another. Host processes often inadvertently assist pathogens that can hijack immune cells or induce endothelial endocytosis. Here we have used transparent zebrafish to visualize how specific host cells and processes contribute to yeast spread *in vivo*. We find that *Candida* is a sophisticated pathogen that uses redundant strategies to disseminate in the host: it can either use host immune cells or cross into the circulatory system and use blood flow. These data suggest that both of these mechanisms must be targeted to limit *Candida* dissemination during infection.

## Introduction

*Candida albicans* is a small non-motile fungus that can cause disseminated candidiasis in immunocompromised populations. This dimorphic pathogen is a normal commensal organism of the mouth, gastrointestinal track, and vagina that causes life-threatening invasive candidiasis in immunocompromised patients. *C*. *albicans* is able to spread from mucosal sites to internal organs in immunocompromised mouse disease models and this is believed to be a primary infection route for this fungus in humans as well [1–4]. Recent work using a tissue-to-bloodstream *C*. *albicans* dissemination model in zebrafish has established that the yeast form is specialized for promoting infection spread [5]. Although we still do not understand how yeast orchestrate this movement, it has been proposed that *C*. *albicans* spreads via phagocyte-dependent or–independent mechanisms [6, 7].

Bacterial and fungal pathogens utilize the host’s immune cells in a “Trojan Horse” mechanism to spread to outlying tissues, stimulating engulfment, surviving within the phagocyte for sufficient time to allow migration away from the infection, then provoking release from the phagocyte. Both *in vitro* and zebrafish disease models have been used to demonstrate how neutrophils [8] and macrophages [9–12] can be vehicles for dissemination for mycobacterium, *Cryptococcus* and *Streptococcus*. Although *C*. *albicans* was previously suspected of being an extracellular pathogen based on *in vitro* challenges, intravital imaging in the zebrafish model has suggested that it can establish an impasse with macrophages that is a prerequisite for migration from the infection site [13].

Independently of phagocyte-driven spread, *C*. *albicans* may be moved directly into the bloodstream from tissues via endocytosis by endothelial cells or paracellular invasion. *C*. *albicans* can cause endothelial cell damage and stimulate endocytosis *in vitro*, suggesting one strategy for invasion through this barrier [3, 7, 14, 15]. Once fungi get into the bloodstream, they can be carried by the circulatory system throughout the host, a process that results in rapid and universal dispersion [16]. Movement of *C*. *albicans* yeast through the endothelial barrier has not yet been studied *in vivo*, largely due to technical limitations in murine models.

To systematically test the importance of these host-mediated mechanisms of yeast spread for *C*. *albicans* infection, we used a recently described zebrafish yolk infection model [5, 17]. This anatomical structure represents a simplified system where translocation through the endothelium and spread throughout the bloodstream can be monitored through longitudinal imaging of both fluorescent fungi and phagocytes. Using high-resolution intravital imaging, we established that both macrophages and neutrophils engulf yeast, while *C*. *albicans* survives within macrophages and can be released far from the site of infection through non-lytic exocytosis. Systematic elimination of both macrophage and neutrophil function, however, resulted in no loss in dissemination frequency or levels. Similarly, elimination of blood flow did not in and of itself result in a reduction in dissemination. When both phagocytes and blood flow were disabled, however, there was a significant decrease in levels of dissemination. Thus, phagocyte-dependent and–independent modes are redundant and the versatility of *C*. *albicans* in using different strategies ensures robust spread of infection.

## Results

### *C*. *albicans* yeast dissemination from localized infection is preceded by innate immune cell recruitment

Pathogen spread within a host is crucial for pathogenesis, but understanding the role of the host in this process has been particularly difficult to quantify in mammalian infection models. Fungal pathogens can disseminate as yeast or hyphae, and recent work using a larval model of zebrafish infection suggests that yeast are specialized for spread from tissue to bloodstream. We sought to use this uniquely tractable model to probe the host’s role in yeast dissemination, postulating that yeast may spread through phagocyte-dependent or–independent strategies.

We first sought to determine if yeast dissemination is passive or driven by explicit fungal-specific determinants. We reasoned that if dissemination were passive it might be triggered by a given level of fungal burden, enhanced by proximity to the vasculature, or even occur with dead yeast. Previous work has shown that different starting inoculums of yeast do not influence dissemination rate [5], which suggested that dissemination frequency is relatively insensitive to inoculum size. To expand upon this previous work, we performed infections with fluorescent neutrophils and/or macrophages, scored for yeast dissemination and phagocyte recruitment to the site of infection by confocal microscopy, and quantified fungal burden in fish from different stages of infection (S1A Fig; for details, see Materials & Methods). We used the yeast-locked NRG1^OEX^ strain for these experiments because this strain disseminates well and can be reliably scored for dissemination for two days post-infection [5]. Consistent with published work, we found high variability in fungal burden levels and no significant differences in fungal burden among groups of recruited and disseminated larvae when measured by standard colony-forming unit assays or fluorescent pixel quantification of confocal Z-stacks (S1B–S1D Fig). This suggests that neither dissemination nor phagocyte responses are driven by the presence of a threshold of fungi or by “passive” mechanisms. Comparing the location of yeast in the yolk with its distance to fluorescent vascular endothelium, we found that spread of infection is neither associated with proximity of the yeast in the infection site to blood vessels nor proximity of the yeast to the yolk edge (S2A–S2F Fig). Finally, we found that dissemination is significantly reduced with killed yeast, suggesting that yeast provide essential cues to mediate their own spread (S3A and S3B Fig). Together, these data suggest that dissemination and immune recruitment are driven by active rather than passive processes.

**Fig 1:**
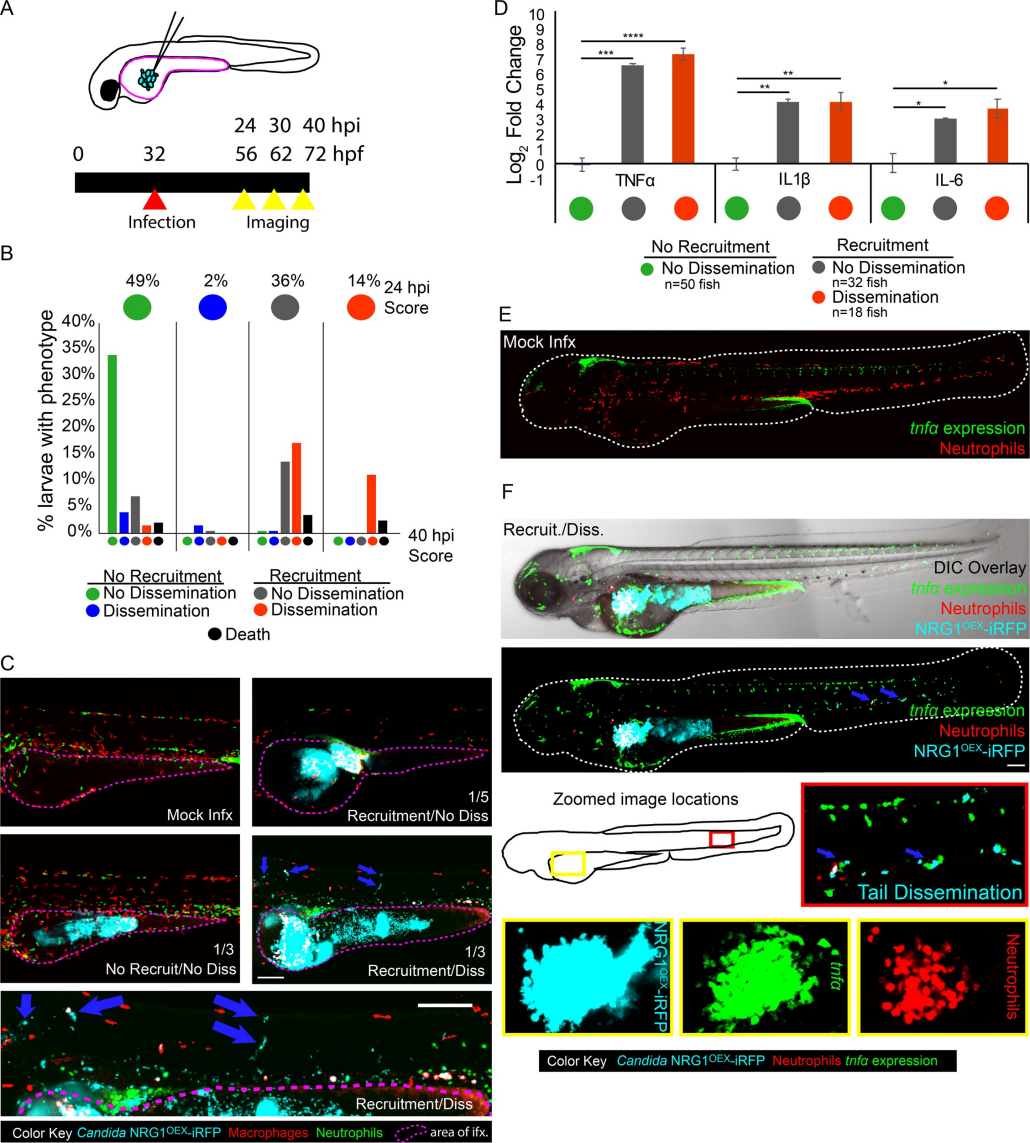
Macrophage and neutrophil recruitment to the site of infection is positively associated with dissemination and proinflammatory cytokine upregulation. Larvae were infected with far-red fluorescent yeast-locked *C*. *albicans* NRG1^OEX^-iRFP in the yolk sac at ~32 hours post fertilization (hpf). (A) Schematic of the yolk sac injection and timeline of the infection and screening for both recruitment of phagocytes and dissemination of yeast. (B) Progression of infection where larvae with early recruitment of macrophages go on to develop disseminated infection and larvae rarely revert back to a non-disseminated state. Top circles represent the percentages of fish, by infection category, at 24 hpi, colored by recruitment/dissemination phenotype. Bottom bars represent subsequent phenotypes at 40 hpi, grouped based on their 24 hpi phenotype. Scores were pooled from larvae with fluorescent macrophages from a total of 12 experiments: 5 experiments in *Tg(mpeg*:*GAL4)/(UAS*:*Kaede)*, 4 experiments in *Tg(mpeg*:*GAL4)/(UAS*:*nfsb-mCherry)*, and 3 experiments in *Tg(fli1*:*EGFP)* x *Tg(mpeg*:*GAL4)/(UAS*:*nfsb-mCherry)*. A total of 197 fish were followed from 24 to 40 hpi. (C) Examples of each infection category in *Tg(mpeg*:*GAL4)/(UAS*:*nfsb-mCherry)/Tg(mpx*:*EGFP)* larvae. The yolk sac is outlined in a dotted magenta line and the approximate proportion of larvae with this score at 40 hpi is indicated in the lower right of the image (the remainder of fish died between 24 and 40 hpi). Arrows indicate disseminated yeast in the representative image of a fish with dissemination, which is also enlarged below for clearer viewing. Scale bar = 150 μm. (D) *Tg(mpeg*:*GAL4)/(UAS*:*nfsb-mCherry)* larvae were scored for macrophage recruitment to the site of infection and yeast dissemination at 24 hpi, then groups of 6–10 were homogenized for qPCR. Gene expression for TNFα, IL1β, and IL-6 were measured and mock-infected larvae were used for reference. Data come from 3 independent experiments with 97 PBS larvae, 50 No recruitment/No dissemination, 32 Recruited/No dissemination, and 18 Recruited/Disseminated total larvae used for analysis. Means are shown with standard error of measurement. One-way ANOVA with Dunnett’s multiple-comparisons post-test demonstrates significantly higher gene expression in larvae with recruited macrophages at the site of infection, regardless of dissemination scores (* p≤ 0.05, ** p ≤ 0.01, *** p ≤ 0.001, **** p < 0.0001). (E-F) Representative *Tg(lysC*:*Ds-Red)/Tg(tnfa*:*GFP)* larvae with red fluorescent neutrophils and green fluorescence with *tnfα* expression in either a mock infected larvae (E) or a yeast-locked *C*. *albicans* infected fish (F). Neutrophils are recruited to the yolk sac and *tnfα* is expressed at the site of infection and in the tail with disseminated yeast cells (indicated with blue arrows). Scale bar = 100 μm.

To understand if host innate immune responses are associated with *C*. *albicans* spread, we infected transgenic zebrafish larvae in the yolk with yeast-locked fungi and intravitally imaged both host immune cells and fungi. Larvae were infected with yeast-locked NRG1^OEX^ (NRG1^OEX^-iRFP; Fig 1A) and scored for recruitment of phagocytes to the infection and yeast cell spread from the infection site. To better understand if immune involvement preceded yeast dissemination or was associated with infection resolution, larvae were longitudinally imaged over a 16-hour time course. To represent this longitudinal data, we grouped individuals by individual score at 24 hpi (designated by the row of colored circles at the top of the graph), then showed progression with lower bars indicating proportions that either stayed the same or changed to a different category (bottom bars showing final phenotypes at 40 hpi). Thus, 49% of fish at 24 hpi had the “No Recruitment/No Dissemination” (NR/ND) phenotype (green circle at top left). These then split into five different categories, where 33% of fish stayed as NR/ND (green bar on far left) while about 5% of fish started as NR/ND at 24 hpi and ended as “No Recruitment/Dissemination” NR/D or “Recruitment/No Dissemination” R/ND by 40 hpi (neighboring blue and gray bars). Fish were overwhelmingly found to be in only three of the four possible classes at 24 hpi, with about 50% in the NR/ND class, almost none in the NR/D class, and 25% each in the other two classes (Fig 1B, top row of colored circles). Larvae that had recruited macrophages at 24 hpi stayed the same or progressed to a recruited state with NRG1^OEX^-iRFP dissemination at 40 hpi. Larvae that began with recruited macrophages and disseminated NRG1^OEX^-iRFP did not revert to a non-recruited or non-disseminated state. In limited cases, dissemination occurred without the response of phagocytes to the yolk sac, and only in one case did it lead to later phagocyte recruitment to the yolk sac. These experiments demonstrated that recruitment of phagocytes tends to precede dissemination (Fig 1B and 1C), suggesting a role for phagocytes in the dissemination of yeast, either directly through the Trojan Horse mechanism, or indirectly through inflammation-mediated weakening of host vasculature [12, 18, 19].

### Phagocyte recruitment correlates with pro-inflammatory gene upregulation and local expression of TNFα

Phagocytes can indirectly facilitate pathogen dissemination by proteolysis of epithelial cell junctions and by producing pro-inflammatory cytokines that increase local vascular permeability [20–23]. We therefore examined if dissemination events were associated with increases in inflammatory cytokine production. We scored for infection spread and recruitment of macrophages and measured cytokine mRNA levels in pooled larvae from each infection category. Larvae that had recruitment of macrophages to the site of infection had significant increases for TNFα, interleukin-1-beta (IL1β), and interleukin-6 (IL-6), and this increase in expression in recruited larvae was independent of dissemination (Fig 1D). We found similar results for infected larvae with both fluorescent neutrophils and macrophages, where increases in TNFα, IL1β, and IL-6 were also independent of dissemination. These data suggest that inflammatory gene expression is associated with phagocyte recruitment, and that cytokine expression at the infection site might precede dissemination.

As TNFα was found to be upregulated in all larvae with phagocyte recruitment, we were interested in determining where TNFα was being expressed in relation to the location of yeast. A transgenic reporter zebrafish line of *tnfa* transcriptional activity was used to visualize the expression of TNFα in *C*. *albicans* infected fish [22]. We found *tnfa*:*EGFP* expression localized primarily at the site of infection and occasionally in phagocytes interacting with disseminated fungi far from the infection site (Fig 1E and 1F). Consistent with the qPCR results, *tnfa*:*EGFP* expression at the site of infection tended to correlate with phagocyte recruitment but not with dissemination (S4 Fig). Taken together, these data suggest that phagocytes recruited to the site of infection produce locally high levels of proinflammatory cytokines that may alter the infection environment, but elevated cytokine levels are not sufficient to drive dissemination events.

### Innate immune cells transport yeast into and throughout the bloodstream

**Fig 2:**
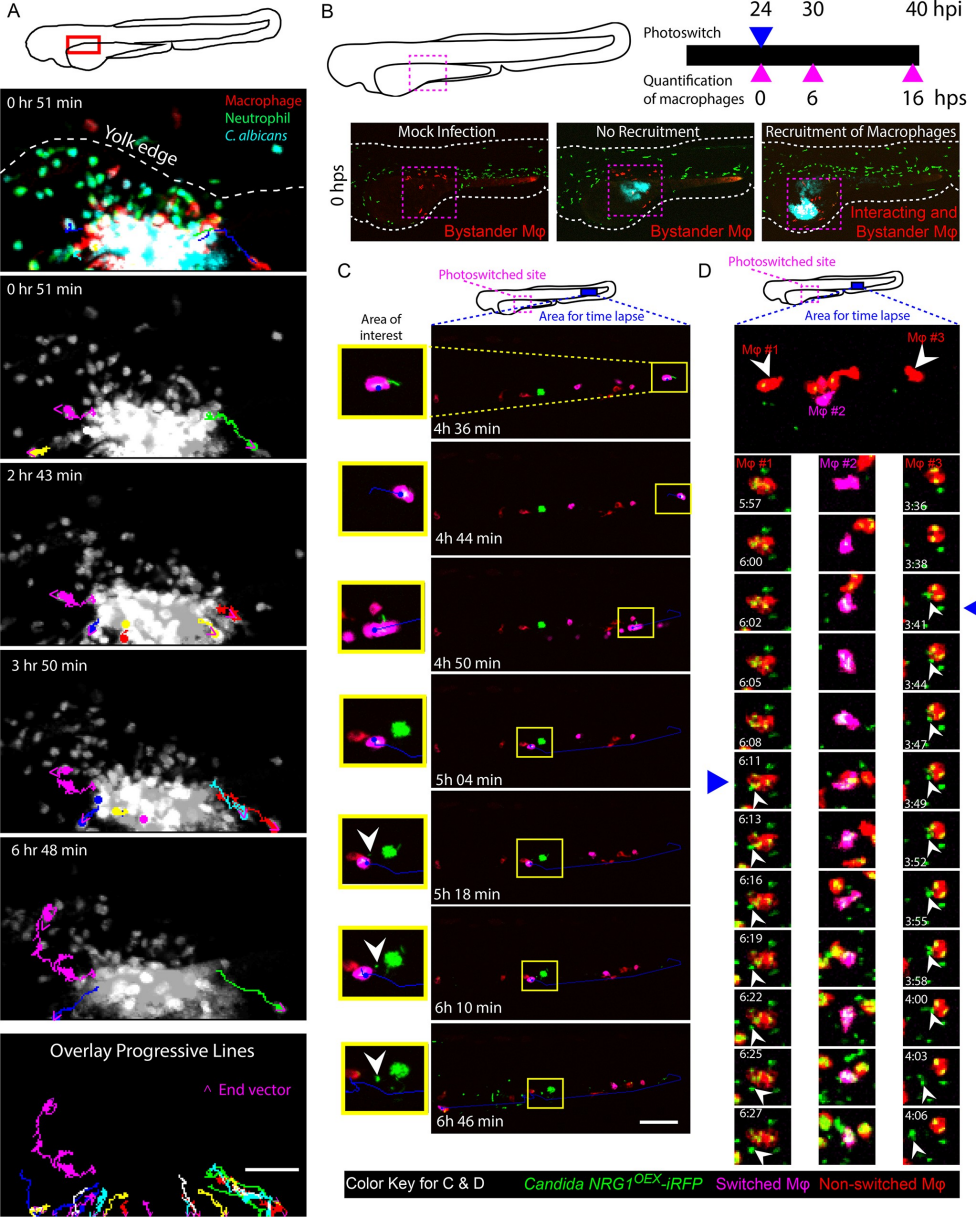
Phagocytes actively participate in the transport of yeast within the bloodstream. *Tg(mpeg1*:*GAL4/UAS*:*nfsb-mCherry)/Tg(mpx*:*EGFP)* or *Tg(mpeg1*:*GAL4)x(UAS*:*Kaede)* larvae were infected with yeast-locked *C*. *albicans* and scored for recruitment of macrophages and dissemination of yeast at 24 hpi. (A) Neutrophils and macrophages were observed moving within and away from the infection site carrying yeast. Time-lapse taken over the course of 7 hours starting at ~32 hpi. The schematic above illustrates the area imaged. The top multicolor image shows the relative distributions of macrophages (red), neutrophils (green) and fungi (blue) and indicates the edge of the yolk with a white dotted line. In lower panels, dots and lines indicate tracks taken by phagocytes during the time lapse, with magenta arrowheads indicating the direction of movement. Phagocytes were observed taking yeast from the site of infection in these cases. The bottom panel illustrates all tracked paths from the time lapse in a single overlay image. Scale bar = 50 μm. Frames taken from S1 Movie. (B) A schematic of our macrophage photo-conversion experiments where *Tg(mpeg1GAL4) x Tg(UAS*:*Kaede)* macrophages near the infection site were photoswitched at 24 hpi, and confocal images of each photoswitched larva were taken at 24, 30, and 40 hpi to track macrophages. Macrophages near the site of infection but not interacting with yeast were identified as “bystander” macrophages. (C) Time-lapse panels of *Tg(mpeg1GAL4) x Tg(UAS*:*Kaede)* larva with a photoconverted macrophage moving out of the blood stream into tail tissue. The top schematic shows the regions used for photoswitching (magenta dashed box) at 24 hpi and the region selected for time-lapse (magenta solid box) from ~46 hpi to ~53 hpi. The yellow highlighted area demonstrates a photo-converted macrophage stopping in the bloodstream, rolling down the tail, and then releasing yeast. White arrowhead in the image panel highlights an apparent non-lytic expulsion (NLE) event. Time stamps indicate HR:MIN. Frames taken from S3 Movie. Scalebar = 100 μm. (D) (Top) Area of tail imaged by time-lapse microscopy in an independent fish from that shown in (C). White arrowheads indicate macrophages followed in detail below. (Below) Middle column of frames highlights yeast growth within a photo-converted macrophage and left and right columns of frames show apparent NLE release of yeast cells from macrophages. Time stamps indicate HR:MIN. White arrowheads within images indicate apparent NLE events. Frames are taken from S4 Movie. Scalebar = 10 μm.

Phagocytes are coopted in the spread of viruses, intracellular bacteria and intracellular fungi [12, 24–28]. Until recent intravital imaging experiments, it was not appreciated that *C*. *albicans* can survive inside phagocytes and create an impasse that could potentially abet its spread into new host tissues through a Trojan Horse mechanism [13]. This “Trojan Horse” mechanism has three distinct stages: first, phagocytes respond to the infection and engulf the pathogen, then phagocytes move out of the infection site, and finally the pathogen exits the phagocyte in distant tissues. To look at overall phagocyte-fungi interaction dynamics in yolk infection, we quantified the number of neutrophils and macrophages at the initial infection site over time. We found that, for fish with fungal dissemination and phagocyte recruitment at 24 hpi, there was an overall decrease in the number of phagocytes at the site of infection over the next 16 hrs. (S5A and S5B Fig; statistically significant difference for macrophages and consistent trend for neutrophils). There was no overall trend for fish that did not have recruitment and dissemination at 24 hpi. This decrease in phagocyte amount at the later time point was either because immune cells were leaving the site or dying as a result of infection [29], but time-course images could not distinguish between these possibilities. Therefore, several time-lapse experiments were performed to examine the fate of macrophages with internalized yeast. These show that macrophages do phagocytose and carry yeast away from the site of infection and into the yolk sac circulation valley, demonstrating a key step of Trojan Horse-mediated dissemination. One example is shown in Fig 2A, where the top panel shows the distribution of phagocytes and fungi at the infection site and shows the large number of both neutrophils and macrophages containing fluorescent yeast. Subsequent panels top to bottom show tracks of individual macrophages containing fluorescent yeast away from the infection site at different times during the time-lapse and then as a composite of all tracked macrophages (Fig 2A; S1 Movie and S2 Movie). These data showed directed movement of macrophages away from the infection site, suggesting a type of reverse migration can take place in this infection model.

Taken together, we saw that phagocytes respond to the infection, phagocytose yeast and carry them from the infection site, but this left open the question of if and how the pathogen escapes from the phagocyte. To follow macrophages from the infection site, we used a transgenic fish with photo-switchable Kaede-expressing macrophages. This enabled longer-term imaging of slow-moving macrophages in a larger cohort of fish by time-course imaging to track them from the infection site to distant sites in the tail. Previous work with this photoconvertible transgenic line showed that macrophages that phagocytose fungi tend to die at the infection site rather than move away [29], but we found that a minority of macrophages with phagocytosed yeast remained viable and migrated from the infection site (Fig 2B). We followed photo-switched macrophages, imaged *C*. *albicans*-macrophage dynamics at 40 hpi in distant infection foci, and found instances of *C*. *albicans* being released from macrophages in each of two larvae within one experiment. In the first larvae, two photoconverted macrophages exocytosed three yeast (Fig 2C and S3 Movie) and in the second larvae one macrophage released a yeast (Fig 2D and S4 Movie). These time-lapses include apparent instances of yeast escaping from macrophages far from the infection site, providing evidence that the third step of the process of phagocyte-aided dissemination does occur *in vivo*.

### Macrophage ablation does not alter overall infection outcomes

While these time-lapse experiments documented the occurrence of Trojan Horse-mediated spread, they are anecdotal and the high-content nature of the experiments prevented quantification of these events in large numbers of infected fish of all cohorts to determine the relative contribution of infected macrophages to overall dissemination. To define the importance of phagocyte-mediated dissemination, we took advantage of the ability to ablate macrophages and disable neutrophils in the zebrafish larva.

We first investigated how much the ablation of macrophages alters yeast spread, eliminating macrophages either by injection of liposomal clodronate [9, 25, 30, 31] or by addition of metronidazole pro-toxin to larvae with nitroreductase-expressing macrophages [32–34]. We confirmed that both methods are effective at eliminating macrophages (S6 Fig). Larvae treated with clodronate liposomes or metronidazole had an almost complete loss of macrophages, and no change in recruited neutrophils or change in fungal burden (S6A–S6F Fig). To our surprise, ablation of macrophages by either method did not result in any alteration in dissemination frequencies at 40 hpi (S7A Fig). Furthermore, ablated larvae also had a similar progression of events where phagocyte recruitment (neutrophils) preceded fungal dissemination (S7B Fig). However, one caveat of the metronidazole experiments is that we did not concurrently treat wildtype larvae to test whether metronidazole itself affects dissemination or progression. Interestingly, there was a trend among disseminated fish towards scores of “High Dissemination” in macrophage ablated fish, when the number of disseminated cells was counted (1–10 = Low, 11–50 = Medium, >50 = High; p = 0.076 for clodronate liposomes and p = 0.103 for metronidazole; S7A Fig). We complemented these chemical ablation experiments with additional experiments using *pu*.*1* morpholinos to block macrophage development. This method also yielded similar dissemination frequencies of yeast with and without macrophage development (S8 Fig). Taken together, these experiments, using three different ways of eliminating macrophages, clearly show that macrophages are unexpectedly not required for efficient dissemination.

### Combined ablation of macrophages and inactivation of neutrophils still does not affect dissemination rates but is associated with increases in extracellular yeast in the bloodstream

**Fig 3:**
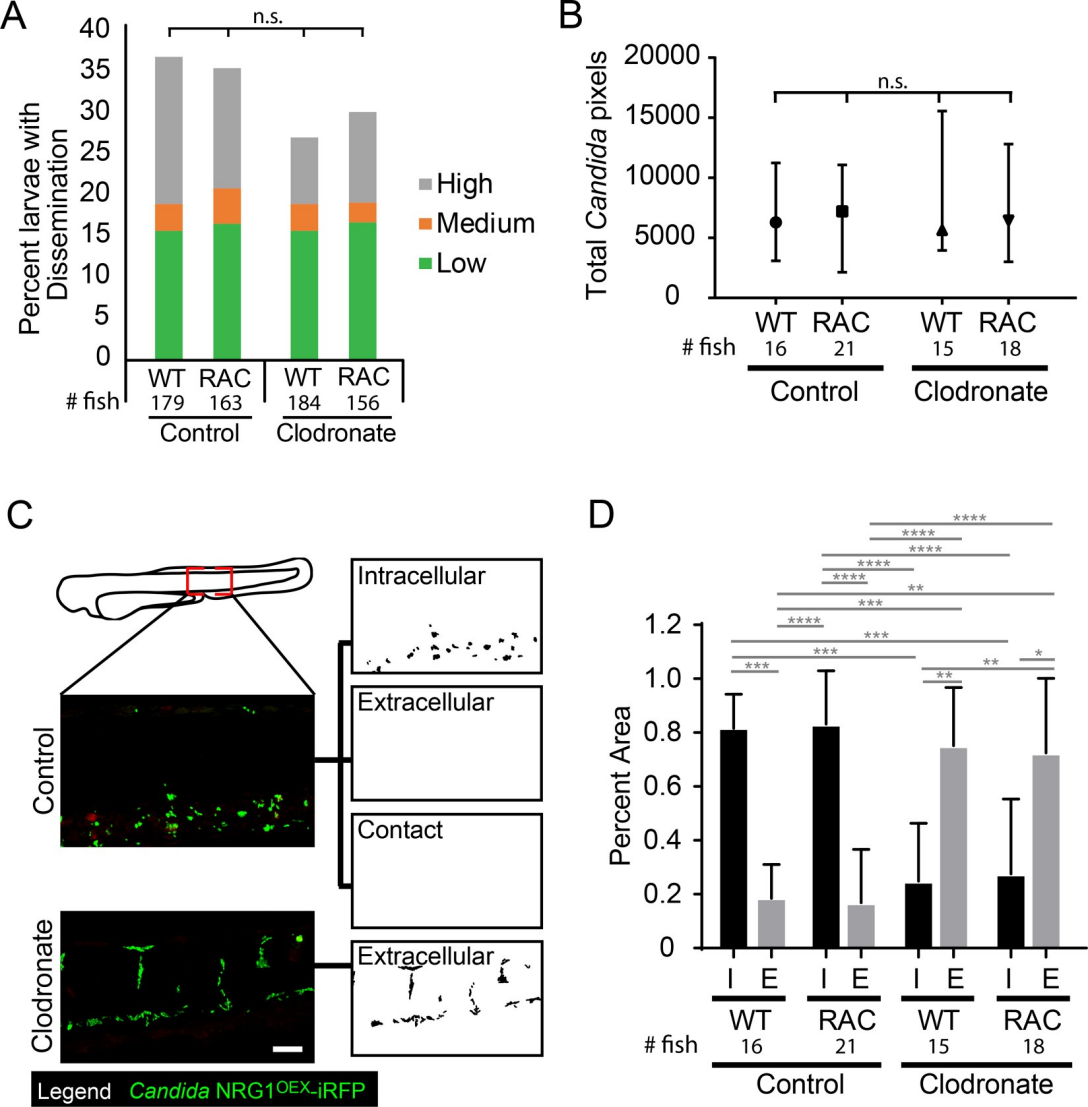
Phagocytes are not required for yeast dissemination to occur. Rac2-D57N and AB sibling larvae were injected with control or clodronate liposomes mixed with a 10 kDa dextran conjugated with Cascade Blue in the caudal vein at 28 hpf. Larvae were infected with the yeast locked *C*. *albicans* 4 hours later as described above. (A) Percent infected larvae with dissemination scored as “low” (1–10), “medium” (10–50), and “high” (>50 yeast) disseminated yeast at 40 hpi. Pooled from 6 experiments. Stats: Fisher’s Exact test, n.s. not significant p>0.05 (B) Total pixel counts for quantified disseminated yeast for each group are shown with the median and interquartile range. Same larvae as in Panels C & D. (C) Method used to quantify disseminated yeast. Yeast were scored as Intracellular (I; inside or in close contact with a phagocyte) or Extracellular (E; not contained or in contact with phagocytes). Images were processed in ImageJ. Scale bar = 50 μm. (D) The proportion of all yeast either Intracellular or Extracellular for each treatment group. Stats: Kruskall-Wallis with Dunn’s posttest (* p≤ 0.05, ** p ≤ 0.01, *** p ≤ 0.001, **** p < 0.0001), and bars indicate the median with interquartile range. Pooled from 6 experiments. Stats: Kruskall-Wallis with Dunn’s post-test. All comparisons were n.s.

Because we observed neutrophils at the infection site and also interacting with disseminated yeast in the tail (S3–S5 Movies), we sought to determine if neutrophils could substitute for macrophages in transporting yeast. We used Rac2-D57N larvae, with defective neutrophils that are unable to extravasate from the bloodstream [35], and eliminated macrophages with clodronate. To our surprise, there was again no difference in dissemination rates in the context of impaired neutrophils, ablated macrophages, or both (Fig 3A). In addition, neither the overall level of dissemination (low, medium or high amount of disseminated yeast; Fig 3A) nor the overall amount of disseminated yeast based on fluorescent pixel counts were significantly altered by phagocyte inactivation (Fig 3B). The fact that virtual elimination of both major phagocyte types does not alter dissemination infection dynamics suggests that Trojan Horse infection spread is a redundant mechanism.

We reasoned that elimination of both major professional immune cell types might alter infection dynamics significantly, limiting the ability of the host to contain bloodstream pathogens and thereby enhancing the ability of yeast to survive and proliferate once gaining access to the bloodstream. We quantified whether disseminated yeast were found intracellularly or extracellularly, as shown in Fig 3C. While larvae without functional macrophages had a higher ratio of extracellular to intracellular yeast, neutrophil perturbation did not affect this ratio (Fig 3D). Together, these results suggest that although phagocytes, particularly macrophages, can participate as Trojan Horses, and macrophages are required to contain disseminated yeast intracellularly, they are not required for dissemination to occur and yeast are able to use other strategies to spread in their absence.

### TNFα production is dependent on macrophages but not neutrophils

**Fig 4:**
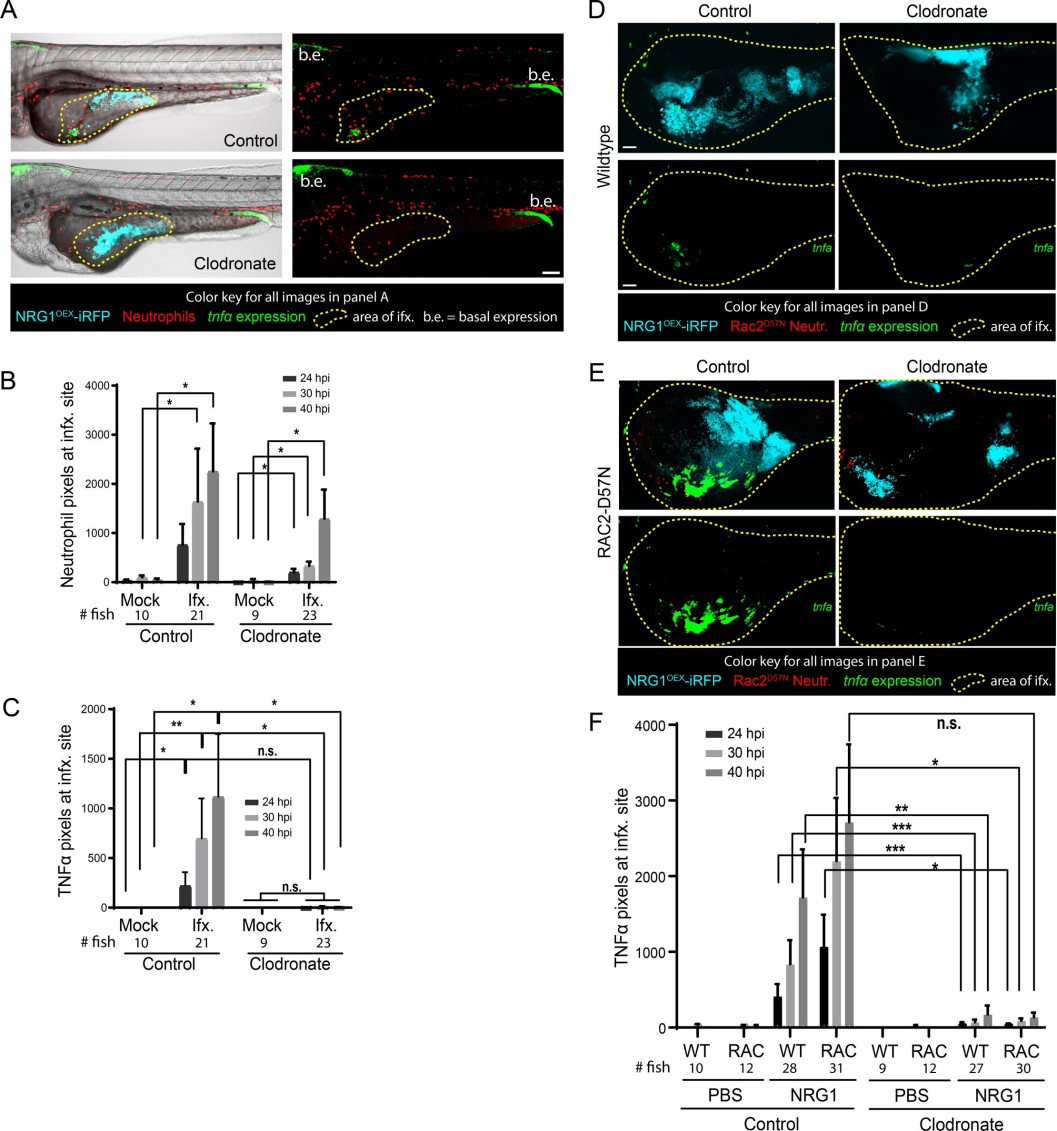
Macrophages are primarily responsible for TNFα production. *Tg(lysC*:*Ds-Red)/Tg(tnfα*:*GFP)* or Rac2-D57N/ *Tg(tnfα*:*GFP)* larvae with red fluorescent neutrophils and green fluorescence with *tnfα* expression were infected with a yeast-locked *C*. *albicans* as described. (A) Representative images of control and clodronate treated *Tg(LysC*:*Ds-Red)/Tg(tnfα*:*GFP)* larvae with functional neutrophils infected with a yeast-locked *C*. *albicans* at 40 hpi. Area in yellow outlines area of *Candida* growth in the yolk sac. Images were used to quantify neutrophil recruitment and *tnfα* expression. Scale bar = 100 μm. Markings are clarified in box below images. (B) and (C) Images were analyzed with ImageJ. (B) Neutrophil recruitment to the area of infection is not affected by macrophage ablation. Data pooled from 5 experiments, total fish used for quantification, left to right: n = 10, 21, 9, 23. Bars are the median with 95% confidence interval. Stats: Kruskall-Wallis with Dunn’s post-test. * p ≤ 0.05. (C) Larvae with ablated macrophages have an almost complete loss of *tnfα* expression. Bars are the means and SEM for ease of viewing. Stats: Mann-Whitney. * p ≤ 0.05, **p ≤ 0.01. (D) and (E) Representative images of control (left) and clodronate (right) treated Rac2-D57N x *Tg(tnfα*:*GFP)* sibling larvae infected with a yeast-locked *C*. *albicans* at 40 hpi. Area in magenta shows the yolk sac outline. Scale bar = 50 μm. (F) Larvae in which macrophages are ablated also have an almost complete loss of *tnfα* expression, which is independent of neutrophils. Pooled from 5 experiments. Stats: Kruskall-Wallis with Dunn’s post-test, * p ≤ 0.05, ** p ≤ 0.01, and bars are the mean and SEM.

These phagocyte ablation data suggested that neither phagocyte type is required for dissemination, but did not address if phagocyte ablation affects local inflammatory gene expression at the site of infection. To determine if *tnfa* expression was altered in response to macrophage ablation, *Tg(LysC*:*dsRed)/TgBAC(tnfa*:*GFP*) larvae with red neutrophils and TNF*a* promoter-driven GFP were infected and imaged for *tnfa* expression in presence or absence of macrophages. Representative images of control liposome- and clodronate liposome-treated larvae are shown in Fig 4A, where the yellow outline depicts the infected area used for analysis. Neutrophils were found at the site of infection in both control and clodronate-treated larvae, but control larvae had slightly more neutrophil recruitment than their clodronate treated counterparts (Fig 4B). TNFα expression was essentially absent in clodronate-treated infected larvae (Fig 4C), correlating with a slight reduction in neutrophil recruitment to the infection site. We sought to determine if there was a relationship between TNFα and neutrophil levels in the clodronate-treated fish, which would indicate if neutrophils drive *tnfa* expression in the absence of macrophages. In control fish, we found a positive correlation between *tnfa*:*EGFP* expression and neutrophil levels, but in the absence of macrophages, this relationship was lost and there was even a slight negative correlation (S9A Fig). Neutrophil recruitment to the site was only slightly reduced in larvae depleted of macrophages, but these larvae demonstrate near total loss of *tnfa*:*EGFP* expression (S9B Fig). This is despite the fact that there is no significant difference in fungal burden among these cohorts (S9C Fig). These experiments suggest that macrophages are the primary drivers of *tnfa* expression at the fungal infection site.

To complement these macrophage experiments, we sought to examine the effects of macrophage ablation in the context of neutrophil inactivation. Rac2-D57N and *TgBAC(tnfa*:*GFP*) fish were crossed to generate larvae with functionally deficient neutrophils and TNFα-controlled green fluorescence. Representative images of control and clodronate-treated fish with median *tnfa*:*GFP* levels at 40 hpi suggest there is no *tnfa*:*GFP* expressed in macrophage ablated fish, which is also apparent in the accompanying Z-stack animations (Fig 4D and 4E, S6–S9 Movies). Quantification of images of these fish reveals a trend toward higher *tnfa* expression in larvae with functional macrophages as compared to the clodronate-treated larvae, regardless of neutrophil status (Fig 4F). Time-lapse movies further demonstrate macrophage-like cells turning on *tnfa* expression (S10 Movie) and the lack of *tnfa* expression in macrophage-ablated fish (S11 Movie). Similar to what was found previously (Fig 3A), there was no change in dissemination rates with loss of one or both phagocytes types in our Rac2-D57N cross with *TgBAC(tnfa*:*GFP*). This data suggests that macrophages are vital for *tnfa* expression either because they are the primary producers of TNFα or because their presence is required so that other host cells are cued to produce TNFα.

### Loss of blood flow reduces overall number of disseminated yeast but does not affect dissemination frequency or tissue location

While many pathogens use phagocytes to traverse epithelial and endothelial barriers, regulated endo- and exocytosis and paracellular movement represent alternative pathways of yeast dissemination into the bloodstream [12, 28, 36]. Extracellular yeast that enter the bloodstream may be then transported away from the infection site by blood flow to distant tissues in the tail or head. To determine the role of blood flow in spreading infection, we took advantage of the ability of zebrafish larvae to grow and develop for the first seven days without a heartbeat, exchanging gases directly with the surrounding water [37–39].

**Fig 5:**
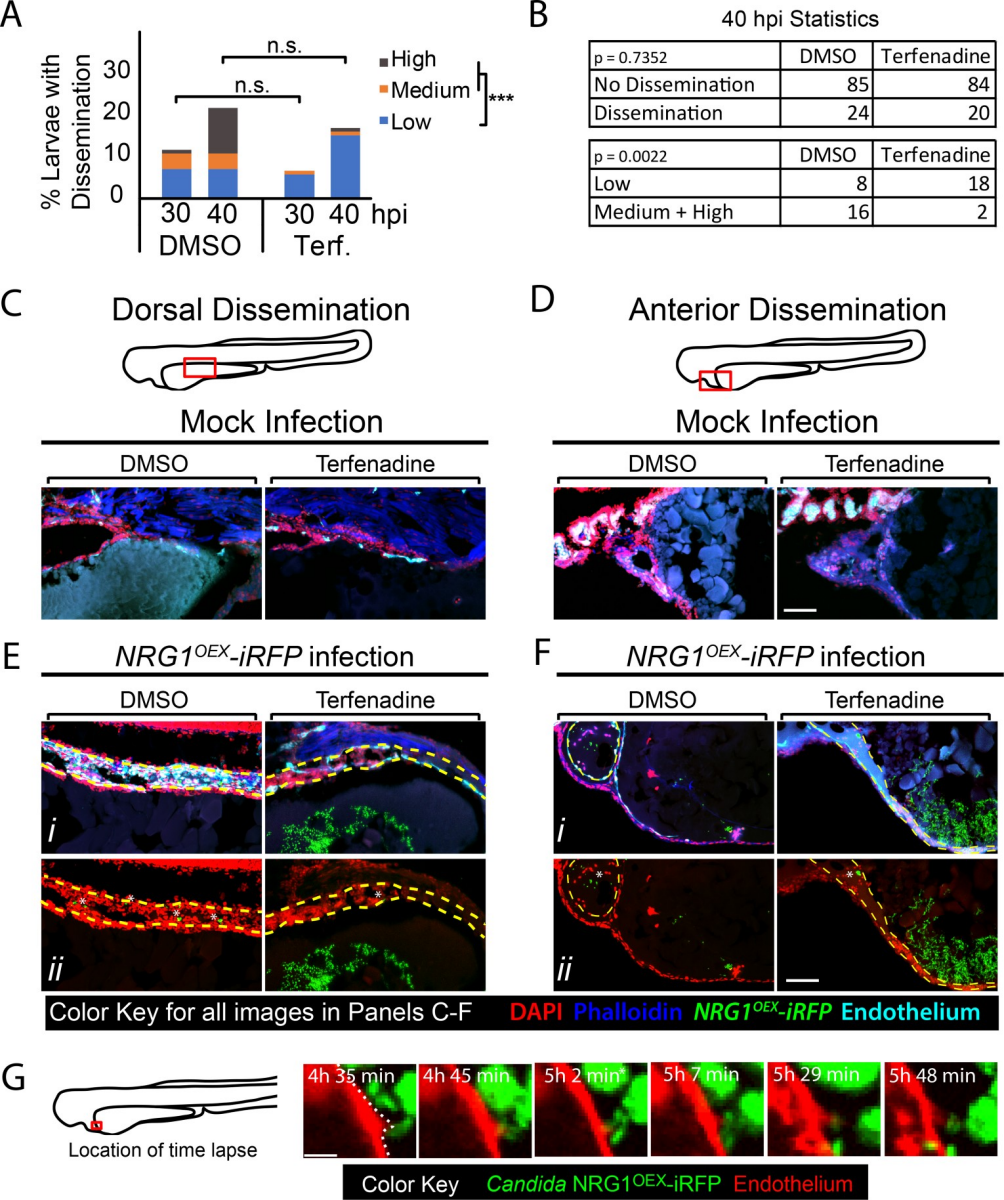
Blockade of blood flow partially limits dissemination. (A-G) *Tg(fli1*:*EGFP)* larvae, with GFP-expressing endothelial cells, were infected as previously described, treated with vehicle (DMSO) or terfenadine (2 μM) and processed for histology at 40 hpi. (A-B) Percent larvae with dissemination at 30 and 40 hpi scored as having “low” (1–10), “medium” (10–50), and “high” (>50 yeast) disseminated yeast. Pooled from 7 experiments, DMSO n = 110, Terfenadine n = 118. Fisher’s Exact test was used to test for differences between “low” and “medium” plus “high” scores at 40 hpi, *** p ≤ 0.001. (C-F) Sections of *Tg(fli1*:*EGFP)* mock infected (C-D) and *Candida*-infected (E-F) larvae compare dorsal dissemination (E) and anterior dissemination (F) events to the same tissue in mock-infected fish. Sections were stained with DAPI (nuclei, red) to indicate surrounding cells and phalloidin (actin; blue) to indicate host structures. NRG1^OEX^-iRFP (green) and *Tg(fli1*:*EGFP)* (cyan) retained fluorescence during sectioning and staining. White asterisks indicate disseminated yeast which appear to be embedded in the yolk syncytial layer and larval heart. Scale bar = 100 μm. (G) A vehicle-treated *Tg(fli1*:*EGFP)* (red) fish infected with NRG1^OEX^-iRFP (green) with intact blood flow was imaged by time-lapse. Area in red box is the focus of the time-lapse, with frames taken from S14 Movie a cropped area of the complete time lapse included as S15 Movie. Scalebar = 10 μm.

We allowed for development until the time of infection to avoid disrupting normal hematopoiesis, then blocked blood flow using non-toxic doses of one of two previously characterized inhibitors of heart beat, terfenadine [40, 41] or valproic acid [42, 43]. Time-course and time-lapse imaging of treated larvae showed that neither drug quantitatively or qualitatively reduced phagocyte responses to *C*. *albicans* challenge, demonstrating that phagocyte recruitment is not dependent on blood flow (S10A and S10B Fig; S12 Movie and S13 Movie; terfenadine- and valproic acid-treatment, respectively). Overall dissemination rates were also unchanged between larvae with and without blood flow in both *Tg(fli1*:*EGFP)* fish (Fig 5A and 5B) and in *Tg(Mpeg*:*GAL4)/(UAS*:*nfsB-mCherry)/Tg(mpx*:*EGFP)* fish (S10C and S10D Fig). However, we noticed a qualitative decrease in dissemination levels, so we further analyzed the data by splitting fish into categories of different levels of dissemination. Interestingly, the proportion of fish with “High Dissemination” was lower with loss of blood flow (1% vs 10%, p<0.001; Fig 5B) and a similar trend was seen in additional but underpowered experiments with fewer fish treated with either terfenadine or valproic acid (for terfenadine 0% vs. 9%, p = 0.0686; for valproic acid 0% vs. 15%, p = 1.000; S10C and S10D Fig). This qualitative alteration in the pattern of dissemination suggested that blood flow is not required for minimal spread but does play a role in further spreading the infection.

To explore further whether blockade of blood flow alters the qualitative nature of yeast dissemination, we sought to characterize the context of fungal spread into the fish at higher spatial and temporal resolution. To enhance spatial resolution and assess the entire tissue context of disseminating yeast, we performed confocal microscopy on frozen sections rather than solely focusing on fluorescently marked cells. Examination of mock-infected fish confirmed that drug treatment caused no overall loss of tissue structure either dorsal or anterior to the yolk (Fig 5C and 5D). Histology of infected fish revealed that yeast that have left the yolk are often found in tissues near the thin endothelial layer surrounding the yolk in both vehicle- and terfenadine-treated larvae with blocked blood flow, either dorsally (Fig 5E) or anteriorly (Fig 5F) of the yolk. The similar tissue locations of yeast with and without blockade of circulation suggest that the dissemination path of yeast near the yolk is not affected by eliminating blood flow. Further, these high-resolution images show that yeast are clearly found within blood vessels with endothelial cells expressing the *fli1*:*EGFP* reporter, pseudocolored in cyan [44]. We also were able to observe the dynamic movement of yeast from the yolk to the blood vessel through time-lapse imaging (Fig 5G, S14 Movie). The frames in Fig 5G demonstrate the movement of yeast through the endothelial cell layer directly from the yolk sac in a vehicle-treated larva. While the movie clearly shows movement into the blood vessel and adherence in the context of strong blood flow, we are unfortunately unable to deduce whether this was host micropinocytosis or paracellular invasion. Nevertheless, given that the movement occurs through an endothelial barrier that appears intact after the migration event, this time-lapse suggests how host endothelial cells and/or phagocytes may play a role in the passage of yeast from a localized infection site to the bloodstream.

### Spread of yeast to distant tissues is inhibited with loss of phagocyte activity and blood flow

**Fig 6:**
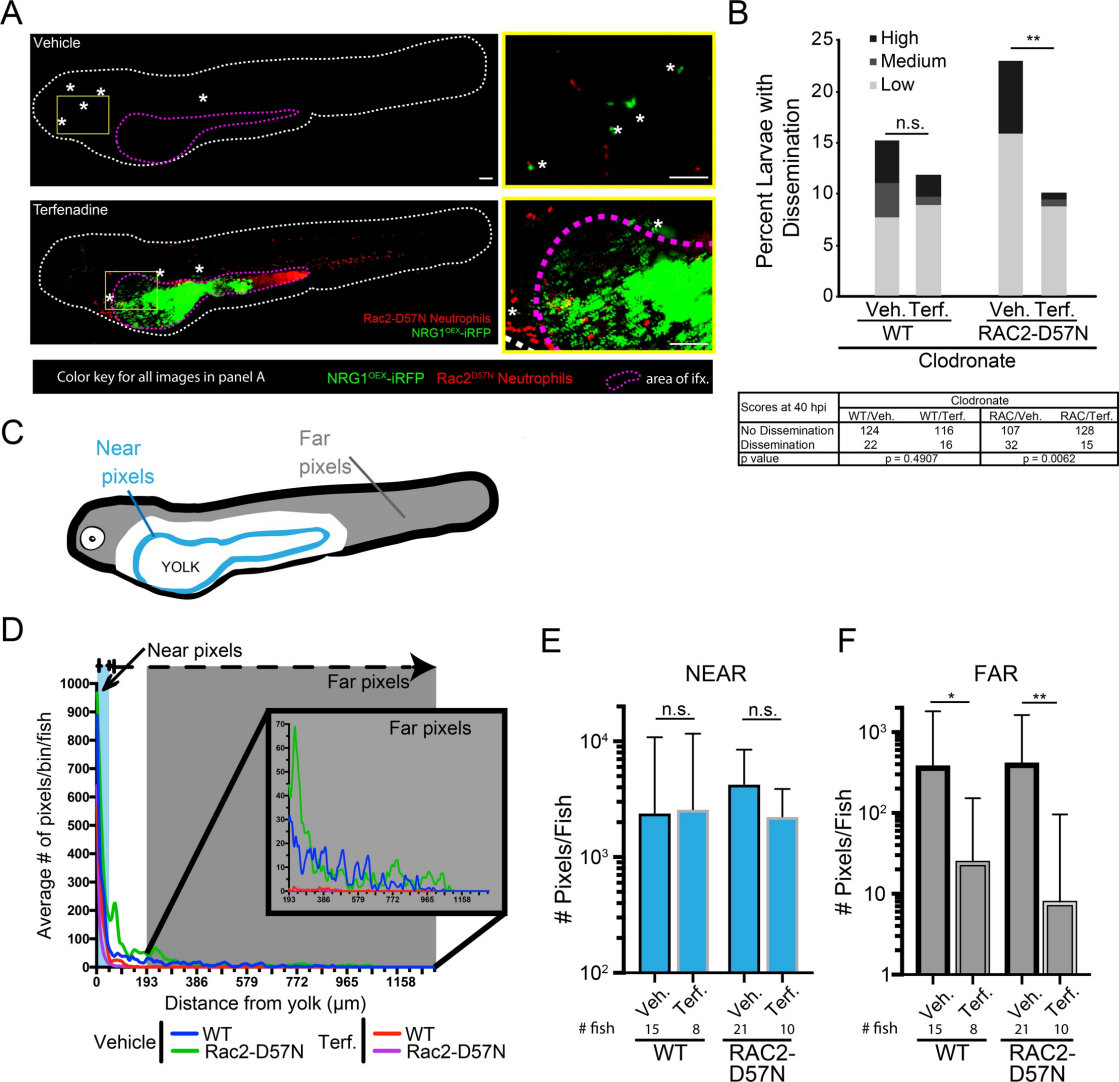
Dissemination is inhibited with loss of phagocytes and blood flow. Rac2-D57N zebrafish were crossed with AB fish for neutrophil-deficient or wild type offspring. All larvae were injected with clodronate liposomes for macrophage ablation and bathed in vehicle (DMSO) or 2 μM terfenadine for blood flow blockade. Larvae were then infected with NRG1^OEX^-iRFP. (A) Representative Rac2-D57N larvae with dissemination either with (Vehicle) or without (Terfenadine) blood flow. The white line outlines the fish body, the magenta line indicates the yolk sac, and white asterisks denote yeast that are disseminated. The yellow box indicates an area with disseminated yeast that has been magnified at the right. Scale bar = 100 μm. (B) Percent larvae with scores of dissemination at 40 hpi, pooled from 6 experiments. Stats: Fisher’s exact test, * p ≤ 0.05. (C) Schematic of the scoring system for disseminated yeast. Yeast that were ≤25 pixels from the yolk sac edge were scored as “near” and yeast that were ≥55 pixels from the yolk sac edge were scored as “far”. (D) A frequency distribution histogram of the distance in pixels that yeast travel from the yolk sac edge sorted into single bins. Distances less than 5 pixels was omitted from analysis. Same fish quantified as in C and D. Pooled data from 54 larvae. (E) Total number of near yeast pixels per fish, shown as median and confidence interval (F) Total number of far yeast pixels per fish, shown as median and confidence interval. (D-F) Pooled from 6 experiments. Stats: Mann-Whitney, n.s. not significant, * p ≤ 0.05, ** p ≤ 0.01.

Blockade of blood flow reduces the number of fungi that leaving the infection area but does not affect the percent of fish with some tissue-to-blood dissemination. Because phagocyte responses were not reduced or eliminated by blocking heartbeat (S10 Fig), we reasoned that phagocytes might account for basal rates of dissemination. To test the combined activity of blood flow and phagocyte-mediated spread, we quantified dissemination in larvae lacking phagocyte activity and heartbeat using *mpx*:*Rac2-D57N* to block neutrophil function, clodronate liposomes to ablate macrophages, and terfenadine to block blood flow. As expected, based on our previous experiments (Fig 4), larvae lose *tnfα* expression with macrophage ablation, which is not correlated with the amount of *Candida* in the yolk (S11 Fig). Rac2-D57N larvae treated with clodronate and terfenadine were found to have disseminated yeast limited to tissues adjacent to the yolk, while disseminated yeast in control larvae were transported to more distant places (Fig 6A). Semi-quantitative scores based on the number of *C*. *albicans* outside of the yolk showed that loss of blood flow did not significantly affect the overall percentages of fish with dissemination, although there was a trend toward less dissemination with blockade of heartbeat (Fig 6B). However, the differences were much larger in the context of neutrophil inactivation, leading to a statistically significant drop in dissemination frequency when comparing phagocyte inactivation alone to phagocyte inactivation in combination with blockade of circulation (Fig 6B). Our inspection of confocal z-stacks suggested that blockade of blood flow reduced the overall distance traveled by yeast. To quantitatively test whether blood flow moved disseminated yeast far from the infection site, we measured this disparity in near (<25 pixels from yolk) versus far (>55 pixels from yolk) dissemination (Fig 6C). Strikingly, there was a significant difference in the pattern of yeast dissemination when macrophages were ablated and blood flow was blocked (Fig 6D). Statistically, there were no significant differences in the number of yeast outside but close to the yolk in any condition (Fig 6E). In contrast, loss of blood flow in the context of macrophage ablation caused a significant decrease in the number of distantly-disseminated yeast (Fig 6F). This loss of extensive dissemination was even more severe when neutrophils were also inactivated (Fig 6F; righthand two bars). Together, these data suggest that a combination of redundant host activities can play important roles in the spread of yeast from the infection site.

### Dissemination of wildtype yeast follows similar pattern as yeast-locked yeast

**Fig 7:**
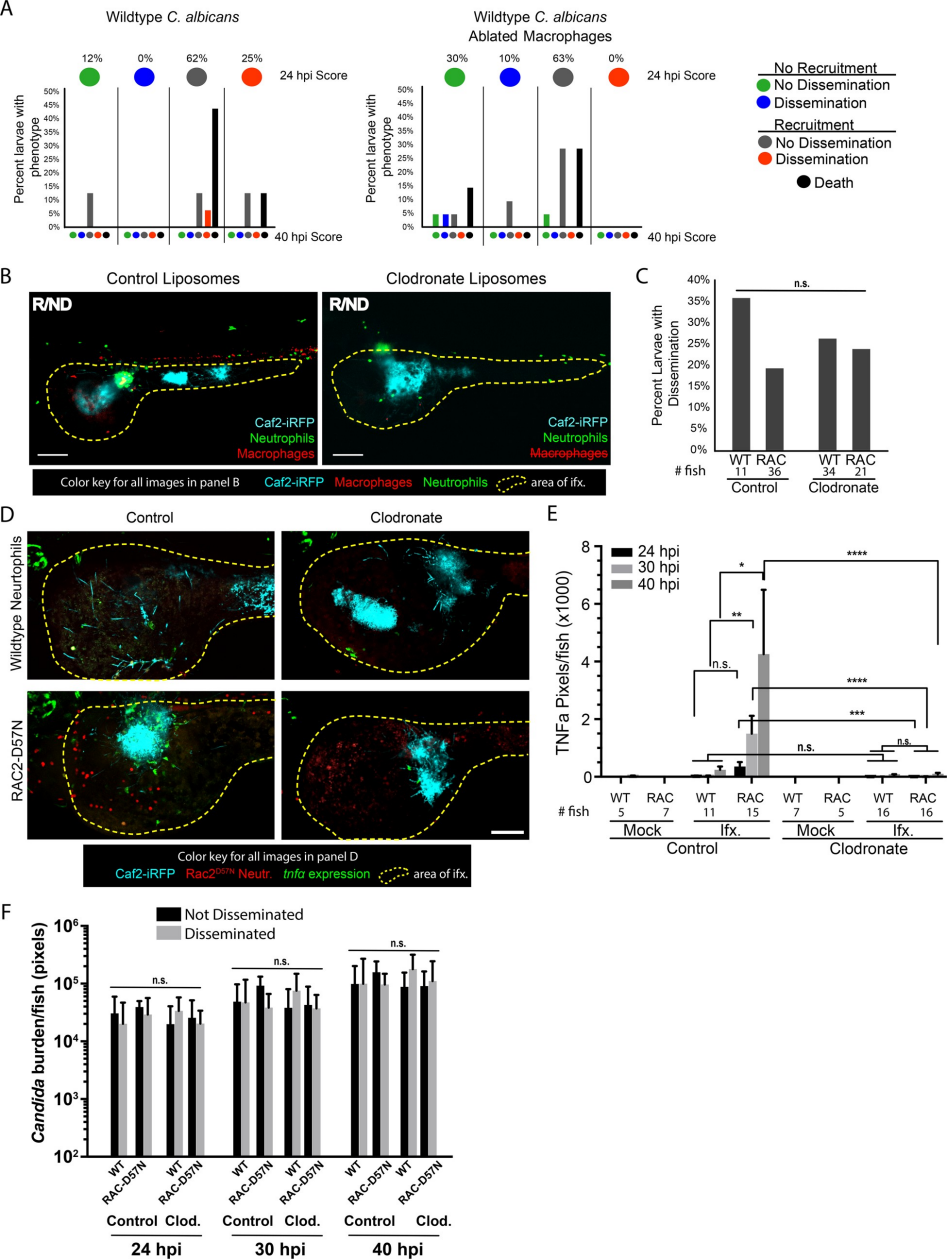
Wild type *C*. *albicans* mirrors yeast-locked strain. *Tg(Mpeg*:*GAL4)/(UAS*:*nfsB-mCherry)/Tg(mpx*:*EGFP)* or RAC2-D57N/*Tg(tnfα*:*GFP)* larvae were injected with control or clodronate liposomes and infected with the wild type Caf2-iRFP *C*. *albicans* as described for the yeast-locked infections. (A) Dissemination dynamics for wildtype fungi are similar to dynamics for yeast-locked and are unaffected by macrophage ablation. *Tg(Mpeg*:*GAL4)/(UAS*:*nfsB-mCherry)/Tg(mpx*:*EGFP)* were infected and scored for immune recruitment and dissemination of yeast between 24 and 40 hpi, as described in Fig 1B. Top circles represent the percentages of fish at 24 hpi, colored with the indicated recruitment/dissemination phenotype. Bottom bars represent phenotypes at 40 hpi, grouped based on their 24 hpi phenotype. Pooled from 2 experiments, control larvae n = 16, clodronate larvae n = 21. (B) Representative images of Recruited/Non-disseminated *Tg(Mpeg*:*GAL4)/(UAS*:*nfsB-mCherry)/Tg(mpx*:*EGFP)* larvae treated with control or clodronate liposomes at 40 hpi. Area in yellow shows the yolk sac outline. Scale bar = 50 μm. (C) Dissemination of wildtype yeast does not require intact phagocytes. Percent dissemination of *Tg(Mpeg*:*GAL4)/(UAS*:*nfsB-mCherry)/Tg(mpx*:*EGFP)* larvae (left) and RAC2-D57N/*Tg(tnfα*:*GFP)* larvae (right) at 40 hpi. Right: Pooled from 2 experiments as in panel A. Left: Pooled from 3 experiments. Fisher’s exact test, n.s. (D) Wildtype elicits a macrophage-dependent expression of TNFα. Representative images of control (left) and clodronate (right) treated Rac2-D57N/*Tg(tnfα*:*GFP)* larvae. Yellow outlines the yolk sac. Scale bar = 100 μm. (E) Pixel area of host cells expressing *tnfα* during wild type *C*. *albicans* infection. Pooled from 3 experiments. (F) Fungal burden quantified as fluorescent pixels in z-stack images (for each time-point, from left to right n = 8, 3, 8, 7, 10, 6, 10 and 6 larvae). Stats: Kruskal-Wallis with Dunn’s post-test, * p ≤ 0.05, ** p ≤ 0.01, *** p ≤ 0.001., **** p ≤ 0.0001, n.s. p > 0.05. Bars indicate the mean and SEM.

These data, taken together, suggest that yeast spread to distant tissues by a combination of phagocyte-mediated dissemination and extracellular dispersal through the epithelial and endothelial layers followed by circulation-mediated movement. While use of the yeast-locked NRG1^OEX^ strain enabled these detailed analyses of dissemination in the absence of extensive tissue damage and early death, we felt it was important to determine if key aspects of yeast dissemination are mirrored during infections with wildtype *C*. *albicans* that produce a mix of filaments and yeast. Infections with wildtype *C*. *albicans*—at a low temperature that promoted production of both yeast and filaments—resulted in the expected high levels of mortality due to invasive filaments [5]. Nevertheless, we were able to confirm that phagocyte recruitment precedes dissemination (Fig 7A and 7B), and dissemination frequency is unaffected by loss of phagocyte function (Fig 7C). Furthermore, phagocyte recruitment to the site of infection is accompanied by a macrophage-dependent local upregulation of TNFα when neutrophils are disabled (Rac2-D57N) but not when neutrophils are functional (Fig 7D and 7E; S15 Movie and S16 Movie). These contributions by neutrophils may reflect neutrophil-hyphal interactions present only with the wildtype *C*. *albicans*. Many interactions of macrophages and neutrophils with fungal cells were captured in these experiments (S15 Movie, S16 Movie and S17 Movie). Consistent with previous work that showed no association of fungal burden with dissemination [5], we found no differences in fungal burden either between fish with and without dissemination or between immunocompetent versus immunocompromised fish (Fig 7F). Taken together, these data suggest that the processes of yeast dissemination are similar for both yeast-locked and wildtype yeast.

## Discussion

Although invasive candidiasis poses a significant clinical risk, we still understand little about how this small non-motile fungus spreads throughout a host and what roles the host itself plays in limiting or enabling its movement. Using longitudinal intravital imaging of *C*. *albicans* and host innate immune cells in zebrafish, we find that phagocyte-dependent and -independent mechanisms provide redundant pathways from tissue to bloodstream and throughout the host. On one hand, we find phagocytes can traffic yeast far from the infection site even in the absence of blood flow, releasing them in distant tissues. On the other hand, we show that even when phagocytes are disabled the yeast are able to efficiently get into the bloodstream and use blood flow to reach far tissues. These multiple strategies emphasize the versatility of *C*. *albicans* and suggest that we need to understand fungal interactions with both endothelial cells and phagocytes to understand the mechanics of tissue-to-bloodstream dissemination. It will be interesting to further test whether this redundancy in host contributions to yeast dissemination holds true in other zebrafish infection routes and in infections with larger hosts such as mammals, experiments which will undoubtedly be enabled by future advancements in our ability to non-invasively image multiple sites over longer periods of time at single-cell resolution in mice.

Phagocyte recruitment correlates with pro-inflammatory gene upregulation and local *tnfα* expression. Cytokine upregulation is a key step in innate immune response to an infectious threat, and this is the case in our infection model [45]. Imaging by time-lapse at the single cell level revealed that *tnfα*-expressing cells are motile and are absent when macrophages are depleted, suggesting that only macrophages express *tnfα* in both yeast-locked and wildtype fungal infections. Although imaging of *tnfα* levels in macrophage transgenics was not performed here, nearly all *tnfα*-GFP+ cells were found to also be *mpeg1*:*mCherry*+ in a similar swimbladder infection model [46]. Proinflammatory cytokines at the infection site likely promote vascular permeability that may enhance fungal spread to the bloodstream by endocytic, paracellular and/or Trojan-horse pathways [7, 22, 23, 47].

We documented all of the key steps of Trojan Horse-mediated spread of *C*. *albicans in vivo*: recruitment, phagocytosis, reverse migration and fungal escape. The hijacking of phagocytes by *C*. *albicans* raises important questions about how and why innate immune cells transport yeast into the bloodstream and release them far from the infection site. While neutrophil reverse migration is now well-documented [48–52], we still do not know what host and fungal signals regulate yeast-laden macrophages to leave the infection site. Further, although non-lytic exocytosis (NLE) is utilized by many pathogens, its regulation is poorly understood and likely involves both host and pathogen cues [24, 53]. Further work focused on NLE *in vivo* may reveal more about how *C*. *albicans* and other pathogens escape macrophage containment during vertebrate infection.

Neutrophils and macrophages were found to be dispensable for efficient dissemination. This was unexpected because we documented Trojan Horse dissemination, we found that phagocytes are sufficient to spread *C*. *albicans* in the absence of blood flow, and we know that macrophages are key for spread of other fungal and bacterial pathogens [24, 53]. One obvious hypothesis is that phagocyte loss enhances fungal burden sufficiently to promote spread by mass action. However, the predictions of this hypothesis are not borne out: our results show that there is not a robust enhancement of burden due to loss of phagocyte activity, and further show for individual infected fish that those with disseminated infection do not have greater burden than those with localized infection (Fig 3D, S9 Fig and S11 Fig). This combination of cohort and individual data are inconsistent with the idea that a threshold of high numbers of yeast in the yolk can non-specifically drive dissemination. Although we would have expected uniformly higher numbers of yeast in fish lacking all phagocyte activity, it is possible that the fungal burdens are comparable because of an overall limited phagocyte response in the context of this localized infection model. *C*. *albicans* may be able to efficiently spread without hitchhiking on phagocytes because it grows relatively well extracellularly compared to other phagocyte hitchhikers and/or because it has effective alternative means of entering and exiting the bloodstream without phagocytes. Further, as phagocytes both limit infection and spread yeast, there may be a counterbalancing effect of phagocyte elimination, where extracellular yeast are able to survive and proliferate in the blood in their absence. As expected in this situation, there is a trend toward some increase in dissemination without recruitment in fish without macrophages but with active neutrophils, suggesting a possibly minor but unique role of macrophages in the process (Fig 7A, 0% vs. 10% are NR/D; S7B Fig, 2% vs. 5% are NR/D; n.s. for both). In this scenario, a decrease in phagocyte-mediated spread could be made up for by an increase in yeast survival and proliferation in the bloodstream in the absence of attacking immune cells.

How do *C*. *albicans* yeast get through the epithelium and endothelium without phagocytes? Hyphae are known to translocate through epithelial and endothelial cell barriers *in vitro*, but yeast translocation has not yet been analyzed in these systems. Yeast passage through epithelial layers is infrequent in most epithelial *in vitro* models of barrier passage, which could be due to the loss of normal tissue architecture *in vitro* and/or to the altered expression of ligands and receptors that mediate internalization. There are a number of candidate host receptors, as well as *C*. *albicans* adhesins and proteolytic enzymes that could participate in yeast translocation, including cadherins, EphA2, EGFR, ErbB2, secreted aspartyl proteases (SAPs), candidalysin, Als3p [7, 54, 55].

Blood flow and phagocyte-mediated dissemination represent redundant mechanisms for *C*. *albicans* to spread throughout the host body from a localized infection. While it had been postulated that both strategies might be used, full-animal imaging of infection progression enabled the quantification of both mechanisms at an unprecedented level. Interestingly, loss of blood flow alone reduces the amount of disseminated yeast within fish with dissemination but not rates of dissemination into the bloodstream or far from the infection site. On the other hand, elimination of phagocyte function alone affects neither overall levels of spread nor frequency. The dissemination of yeasts adjacent to the yolk in the absence of heart beat and phagocyte function suggests that diffusion through blood vessels isn’t sufficient to carry away yeast, perhaps because of adherence to the vascular lumen [56, 57]. It is intriguing that the two mechanisms are not additive, highlighting the potentially double-edged sword of phagocytes that can enhance dissemination through Trojan horse-mediated mechanisms but can also limit spread of free yeast through phagocytosis and containment. The higher level of dissemination and greater amount of extracellular yeast we observe in phagocyte-incapacitated hosts (Fig 3 and S7 Fig) is consistent with an important role for phagocytes in limiting fungal proliferation in the bloodstream. Tracking the fate of individual phagocytes by adapting the Zebrabow fish line [58, 59] and/or imaging individually photoswitched yeast should allow single-cell quantification of these activities in the future.

While the larval zebrafish model has unique advantages in imaging and manipulation, these come with some limitations as well. The small size of the fish allows one to image the whole fish on the confocal, but the related limitation of the model is that dissemination distances are quite small, compared to what you would have in a mouse or human. Therefore, in translating our findings to the human host, we may find a differential time frame for extracellular spread by rapid blood flow as compared to phagocytes moving more slowly through tissue and lymph. Furthermore, although the overall anatomy is similar between fish and humans, the tissue architecture of yolk, epithelium and endothelium is not representative of all potential sites of tissue-to-bloodstream dissemination in mammals. Based on our findings in the zebrafish and the known conservation of cell types and molecules among vertebrates, we predict that both phagocyte-mediated and extracellular mechanisms of spread are important in mammalian tissue-to-blood dissemination. Unfortunately, although large groups of fungi can be tracked non-invasively in the mouse, testing of these ideas in a mammal will have to wait for new methods that will allow tracking of individual yeast and phagocytes to distant tissues [60–67].

Leveraging the advantages of the zebrafish model system, we have quantified two redundant means of *C*. *albicans* yeast spread from tissue to blood. We documented Trojan Horse spread of *C*. *albicans* and showed that it is redundant with phagocyte-independent dissemination. Conservation of adhesion molecules, cell types and anatomy among all vertebrates suggests that both mechanisms are likely to also be important in mammalian infection [68–71]. Clinical observations are consistent with our findings that *C*. *albicans* moves in the blood both inside and outside of phagocytes [72–75]. From a therapeutic standpoint, our results suggest that prevention of *C*. *albicans* dissemination will require interventions that block both endothelial and phagocyte-driven movement of yeast.

## Materials and methods

### Ethics statement

All zebrafish studies were carried out in accordance with the recommendations in the Guide for the Care and Use of Laboratory Animals of the National Research Council [76]. All animals were treated in a humane manner and euthanized with Tricaine overdose according to guidelines of the University of Maine Institutional Animal Care and Use Committee (IACUC) as detailed in protocols A2015-11-03 and A2018-10-01.

### Animal care and maintenance

Adult zebrafish used for breeding were housed at the University of Maine Zebrafish Facility in recirculating systems (Aquatic Habitats, Apopka, FL). All zebrafish studies were carried out in accordance with the recommendations in the Guide for the Care and Use of Laboratory Animals of the National Research Council [76]. All animals were treated in a humane manner and euthanized with Tricaine overdose according to guidelines of the University of Maine Institutional Animal Care and Use Committee (IACUC) as detailed in protocols A2015-11-03 and A2018-10-01. Following collection, embryos were kept 150 mm petri dishes with E3 media (5 mM sodium chloride, 0.174 mM potassium chloride, 0.33 mM calcium chloride, 0.332 mM magnesium sulfate, 2 mM HEPES in Nanopure water, pH 7) plus 0.3 mg/liter methylene blue (VWR, Radnor, PA) to prevent microbial growth for the first 6 hours. Larvae were then moved to fresh E3 media supplemented with 0.02 mg/ml of 1-phenyl-2-thiourea (PTU) (Sigma-Aldrich, St. Louis, MO) to prevent pigmentation. Larvae were reared at 28°C at a density of 150 larvae per 150 mm petri dish. Transgenic lines used are provided in Table 1.

**Table 1:** Zebrafish lines used.

Transgenic line	Reference
*Tg(mpx*:*EGFP)i11rTg*	[77]
*Tg(mpeg1*:*GAL4)gl24Tg*	[34, 78]
*Tg(UAS-E1b*:*NTR-mCherry)c264Tg*	[79]
*Tg(Mpeg1*:*GAL4)/(UAS*:*Kaede)*	[32]
*TgBAC(tnfa*:*GFP)pd1028Tg*	[22]
*Tg(LysC*:*dsRed)*	[22, 80]
*Tg(fli1*:*EGFP)*	[44]
*Tg(mpx*:*mCherry*,*rac2_D57N)zf307Tg*	[35, 81]
AB (wild type)	Zebrafish International Resource Center

### *C*. *albicans* strains and growth conditions

*C*. *albicans* strains used for larval infection (Table 2) were grown for 24 hours at 37°C on yeast-peptone-dextrose (YPD) agar (20 g/L glucose, 20 g/L peptone, 10 g/L yeast extract, 20 g/L agar, Difco, Livonia, MI). Single colonies were picked to 2 x 5 mL liquid YPD and grown overnight at 30°C on a wheel. Prior to microinjection, liquid cultures were washed twice in phosphate buffered saline (5 mM sodium chloride, 0.174 mM potassium chloride, 0.33 mM calcium chloride, 0.332 mM magnesium sulfate, 2 mM HEPES in Nanopure water, pH = 7) and the concentration of yeast adjusted to 5x10^6^ CFU/ml in PBS for larval zebrafish infections. Wild type Caf2-dTomato *C*. *albicans* was used for heat killing and UV inactivating experiments. Overnight cultures were washed twice and resuspended in PBS at 2.5x10^7^ cells/ml, then boiled for 10 minutes or placed in an uncovered polystyrene petri dish (60 mm x 15 mm, VWR, Radnor, PA) for exposure. A CL-1000 UV cross-linker was used for UV inactivation of *C*. *albicans* [82] (UVP, Vernon Hills, IL) and yeast were exposed four times to 100,000 μJ/cm^2^ with swirling between each exposure. Following boiling or UV-inactivation, cells were stained with AlexaFluor 555 by coincubation of cells in PBS with sodium bicarbonate (0.037 M final concentration, pH 8.2) in the dark for 40 minutes with periodic vortexing. Cultures were then washed four times in PBS and brought to 2x10^7^ CFU/ml for injection into the yolk sac. Proper heat killing and UV inactivation was confirmed by plating 50 μl of the prepared 2x10^7^ CFU/ml killed yeast and 50 μl of the prepared 5x10^6^ CFU/ml live yeast used for larval injection on YPD plates and incubated overnight at 30°C.

**Table 2:** *Candida albicans* strains used.

*C*. *albicans* Strain	Parent Strain	Figure	Genotype	Reference
NRG1^OEX^-iRFP	THE21	All other experiments	*ade2*::*hisG/ade2*::*hisG ura3*::*imm434/ura3*::*imm434*::URA3*-* tetO *ENO1/eno1*::ENO1 tetR-ScHAP4AD-3XHA-ADE2 pENO1-iRFP-NATR	This study, [5, 13]
*efg1Δ*/Δ *cph1Δ*/Δ-iRFP	CAI4	S8 Fig	*ura3*::*1 imm434/ura3*::*imm434 cph1*::*hisG/cph1*::*hisG efg1*::*hisG/efg1*::*hisG-*URA3*-hisG* pENO1-iRFP-NATR	[5, 83]
*efg1Δ*/Δ *cph1Δ*/Δ-dTomato	CAI4	S5 Movie	*ura3*::*1 imm434/ura3*::*imm434 cph1*::*hisG/cph1*::*hisG efg1*::*hisG/efg1*::*hisG-*URA3*-hisG* pENO1-dTomato-NATR	[5, 83]
NRG1^OEX^-dTomato	THE21	Fig 1B	*ade2*::*hisG/ade2*::*hisG ura3*::*imm434/ura3*::*imm434*::URA3*-* tetO *ENO1/eno1*::ENO1 tetR-ScHAP4AD-3XHA-ADE2 pENO1-dTomato-NATR	[5, 83]
NRG1^OEX^-neon	THE21	Fig 3	*ade2*::*hisG/ade2*::*hisG ura3*::*imm434/ura3*::*imm434*::URA3*-* tetO *ENO1/eno1*::ENO1 tetR-ScHAP4AD-3XHA-ADE2 pENO1-neon-NATR	
Caf2-dTomato	CAF2.1	S3 Fig	*Δura3*::*imm434/ URA3* pENO1-dTomato-NATR	[84]
Caf2-iRFP	CAF2.1	Fig 7, S15 Movie, S16 Movie and S17 Movie	*Δura3*::*imm434/ URA3* pENO1-iRFP-NATR	[82]

### Zebrafish larvae infection, scoring for dissemination and recruitment, and fungal CFU determination

Larvae at ~32 hours post fertilization were manually dechorionated and anesthetized in fresh E3 media plus 0.02 mg/ml PTU and Tris-buffered tricaine methane sulfonate (160 μg/ml; Tricaine; Western Chemicals, Inc., Ferndale, WA). Larvae were injected in the yolk sac with 5 nl volume PBS control or *C*. *albicans* at 5x10^6^ CFU/ml in PBS. Larvae were microinjected in the yolk as described previously [5] and screened on a Zeiss Axio Observer Z1 microscope to ensure the correct injection and starting inoculum of *C*. *albicans* in the yolk (Carl Zeiss Microimaging, Thornwood, NJ). Properly infected larvae were then moved to fresh E3 media plus PTU which was changed every other day. Where indicated, media was supplemented with metronidazole, terfenadine, or valproic acid following infection. Larvae were kept at 28°C for NRG1^OEX^ yeast locked infections [83] or 21°C for wild type Caf2 infections [5]. These temperatures were chosen as they are safest for zebrafish and allow for yeast and hyphal growth of *C*. *albicans* [5, 78]. Throughout the study, fish were scored by epifluorescence imaging for phagocyte recruitment to the yolk at the site of infection and for levels of dissemination using the scale: 1–10 yeast (low), 11–50 (medium), >50 (high). At 42 hours post infection, larvae were euthanized by Tricaine overdose. Mortality was observed as loss of heartbeat in all experiments where blood flow was not altered. In experiments utilizing valproic acid or terfenadine to reduce heart rate and blood flow, mortality was scored as larval putrefaction (loss of tissue integrity, graying of tissue, and sloughing of dermis layer).

For CFU quantification, groups of 5 larvae were taken after injection of *C*. *albicans* and screening and homogenized at 0 hpi in 600 ul of PBS. 100 μl of the homogenate was plated on YPD agar supplemented with gentamicin (30 μg/ml, BioWhittaker, Lonza), penicillin-streptomycin (250 μg/ml, Lonza), and vancomycin hydrochloride (3 μg/ml, Amresco, Solon, OH). Individual larvae were homogenized for CFUs in 600 μl PBS after the 40 hpi timepoint, and 100 μl of the 1:6 dilution or 1:60 dilution was plated on YPD for countable colonies. Plates were labeled by plate wells so that the larvae CFU count could be paired with dissemination scores and larval images. YPD plates were incubated overnight at 30°C and colonies counted the following day.

### RNA isolation and qPCR analysis

Larvae were pooled into groups based on phagocyte recruitment to the yolk sac and dissemination of yeast (non-recruited/non-disseminated, non-recruited/disseminated, recruited/non-disseminated, and recruited/disseminated). Larvae were euthanized by overdose in tricaine and immediately homogenized in TRIzol (Invitrogen, Carlsbad, CA) for RNA isolation. Larval groups ranged from 2–15 fish in 3 independent experiments. RNA was isolated using the Direct-zol RNA miniprep kit (Zymo Research, Irvine, CA) using the manufacturers recommended protocol. Final RNA was eluted in 20 ul nuclease free water and stored at -80°C until cDNA synthesis. Using the iScript reverse transcriptase supermix (Bio-Rad, Hercules, CA), cDNA was synthesized from 500 ng RNA per sample. Primers are described in Table 3. RT-qPCR was done on a CFX96 thermocycler (Bio-Rad, Hercules, CA) using the cycles: 95°C for 30 s, 95°C for 5 s followed by 60°C for 20 s for 39 cycles, then 95°C for 10 s followed by 65°C for 5 s [82]. The Bio-Rad CFX Manager software was used to analyze threshold cycles and dissociation curves. Larval gene expression was normalized to the *gapdh* control gene (ΔCT), as done previously [82, 84], and compared to the mock infected PBS controls (ΔΔCT).

**Table 3:** qPCR Primers.

Gene	Sequence (5’-3’)	Reference
IL1β	Forward: GTCACACTGAGAGCCGGAAG	[84]
	Reverse: TGGAGATTCCCAAACACACA	
IL6	Forward: GGACGTGAAGACACTCAGAGACG	[82]
	Reverse: AAGGTTTGAGGAGAGGAGTGCTG	
TNFα	Forward: CGCATTTCACAAGGCAATTT	[82, 84]
	Reverse: CTGGTCCTGGTCATCTCTCC	
gapdh	Forward: TGGGCCCATGAAAGGAAT	[82, 84, 85]
	Reverse: ACCAGCGTCAAAGATGGATG	

### Confocal fluorescence microscopy, time-lapse imaging and photoswitching

For imaging, fish were anaesthetized with Tricaine then immobilized in 0.5% low-melting-point agarose (Lonza, Switzerland) in E3 containing Tricaine and arranged in a 24-well glass-bottom imaging plate. Images were made on an Olympus IX-81 inverted microscope with an FV-1000 laser scanning confocal system (Olympus, Waltham, MA), using a 20x/0.7 NA or 10x/0.4 NA objective lens. Fluorescent channels were acquired with optical filters for 635 nm excitation/668 nm emission, 543 nm excitation/572 nm emission, and 488 nm excitation/520 nm emission, for far red fluorescent cells, red fluorescent cells, or green fluorescent cells, respectively. Phalloidin-568 was captured with the optical filter for 543 nm excitation/668 nm emission for histology microscopy. Cascade blue was captured using the optical filter 405 nm excitation/422 nm emission for larvae treated with control or clodronate liposomes.

Time lapse imaging for all experiments was done between 32 and 40 hpi unless noted otherwise. For photoswitching experiments, one larva was chosen for time lapse imaging between 32–40 hpi, between 46–54 hpi, and between 56–64 hpi. Larvae were removed from the initial 24-well plate and re-embedded in an 8-well μ-slide insert for the ibidi heating system K-frame (ibidi, Deutschland) for temperature control. The heating system was set up at least one-half hour before starting the time lapse. Time lapses were run on “FreeRun” and images were acquired in succession. Time lapse imaging was done with a 20X (0.75 NA) objective unless noted otherwise. Individual fluorescent channels were compiled on Fluoview software before being transferred for compilation and analysis in Fiji-ImageJ [86].

For photoswitching, *Tg(mpeg1*:*GAL4)* adults were crossed with *Tg(UAS*:*Kaede)* for larvae with photoconvertible green to red macrophages [78]. Larvae were reared and infected with yeast-locked *C*. *albicans* as described, and at 24 hpi larvae were embedded in 0.5% low melting point agarose (LMA) in E3 media plus Tris buffered tricaine methane sulfonate (160 mg/ml) in glass-bottom 24-well imaging dishes. Larvae were scored for recruitment of macrophages and yeast dissemination prior to photoswitching. Photoswitching was done using a 405 nm laser at 10% power on a Olympus IX-81 inverted microscope with an FV-1000 confocal system for 10 minutes on Fluoview XY repeat with a 20X (0.75 NA) objective [13, 29]. A 10X (0.40 NA) z-stack image was taken of the yolk sac area immediately following photoswitching and of the whole fish at 30 and 40 hpi. Imaging was done at room temperature and larvae were kept at 28°C (yeast-locked infections) or 21°C (wild type infections) between imaging sessions. To keep the fish from drying out, 2 mL of E3 media plus PTU was layered over each larva following the first imaging session.

### Fiji-ImageJ and MATLAB image analysis

Images were processed in Fiji-ImageJ [86] for counting of phagocytes using Z stack projections (Maximum Intensity Projection), image compilation (Maximum Intensity Projection of the Z stack), and mask compilation. Fiji-ImageJ was also used to track phagocyte movement using the Manual Tracking plugin (Fig 2). To quantify the amount of TNFα expressing cells or neutrophils in the area of *C*. *albicans* cells, masks were made of each fluorescent channel (TNFα, neutrophils, and *C*. *albicans*) in Fiji-ImageJ and run through a script in MATLAB (The MathWorks, Inc., R2017b, Natick, MA). MATLAB workflows are described in S12 Fig. The MATLAB script (S1 MATLAB Script; New_TNF_express) quantified the number of TNFα or neutrophil pixels in the area of *C*. *albicans* pixels (Figs 4 and 7, S9 Fig and S11 Fig). It also measured the number of fluorescent pixels in the image, so *C*. *albicans* growth could be measured over time. Note that these calculations were made for individual confocal z-slices and not from maximum projections.

To measure the distance that yeast travelled in Fig 6D and 6F, two MATLAB scripts were used. First, distances for each disseminated pixel were measured using one script (S2 MATLAB Script; Allison_candida_amounts_used). Next, distances were binned by 1-pixel increments and compiled into a single file using the script (S3 MATLAB Script; New_real_Bins_used). Note that these calculations were made for individual confocal z-slices and not from maximum projections. In addition, the data from Allison_candida_amounts_used were also used by the script (S4 MATLAB Script; Allison_combining_candida_amounts_used) to quantify the total level of burden in the yolk and the total amount disseminated. Briefly, masks were made for each fish using the DIC channel to outline the yolk sac and body of the fish. ImageJ masks for the *Candida*, yolk, and body of the fish were read into MATLAB and converted into MATLAB masks for each fish. The amount of *Candida* pixels in the yolk were summed. New *Candida* masks were made to show only disseminated *Candida* pixels and the amount of disseminated *Candida* pixels summed. Distance maps were created from yolk masks giving pixel distances from the yolk. The distance maps and disseminated *Candida* masks were used to find the distances from the yolk for each pixel of *Candida*. The sums of *Candida* in the yolk and disseminated *Candida* as well as the distances for disseminated *Candida* were saved as MATLAB files for each fish to be run through another MATLAB script. The *Candida* sums for the yolk and disseminated *Candida* for each fish were ran through another script to combine the data for each fish into one spreadsheet for *Candida* amounts in the yolk and another for disseminated *Candida* amounts and exported. The *Candida* distances for each fish were run through another script. For each fish the distances were binned so that each bin contained the number of *Candida* pixels at each distance in 1-pixel intervals. The fish were sorted into their respective groups and the bins summed for each group. The groups were then combined into one array and exported into a spreadsheet.

To score and quantify the location (intra- vs. extra-cellular) of disseminated yeast in Rac2-D57N fish treated with clodronate (Fig 3), 20X images were taken on the Olympus FV1000 confocal at 40 hpi. Fiji-ImageJ was used to score the larvae for yeast that was intra- and extracellular while Photoshop (version CS5 12.1 x64, Adobe Systems Incorporated) was used to trace over yeast scored as intra- and extracellular by methods previously described from our laboratory [5, 13]. Photoshop layers were annotated and the area calculated in ImageJ.

### Macrophage ablation

Where indicated, macrophages were ablated by injection of clodronate liposomes (Clodronate liposomes, Amsterdam, The Netherlands) in the caudal vein, larvae were bathed in metronidazole, or embryos were injected with *pu*.*1* morpholino oligonucleotides (MO, Genetools). Liposomes were injected in the caudal vein at a 3:1:1:1 ratio of 5 mg/ml liposome stock:PBS:phenol red:10 kDa Cascade blue dextran (8% w/v) in a total volume of 8–10 nl per larvae [9] at ~28 hpf. Larvae recovered in E3 media for 4 hours and were then infected with *C*. *albicans*. *Tg(Mpeg*:*GAL4)/(UAS*:*nfsB-mCherry)* larvae were bathed in 20 mM metronidazole for 4 hours after injection with *C*. *albicans* yeast and then kept in E3 media plus PTU and 10 mM metronidazole for the remainder of the experiment [34, 78, 87]. Translational blocking (CCTCCATTCTGTACGGATGCAGCAT) and splice blocking (GGTCTTTCTCCTTACCATGCTCTCC) MOs were co-injected into 1–2 cell stage embryos [10, 30]. The MOs were combined (2.5 ng translation blocking and 0.4 ng splice blocking) with 10 kDa Cascade blue dextran (8% w/v) and phenol red for visualization of injection with total volume injected at 2 nl. Ablation of macrophages with MO technology was observed by loss of red fluorescence in *Tg(Mpeg*:*GAL4)/(UAS*:*nfsB-mCherry)* larvae.

### Chemical blockade of blood flow

For chemical inhibition of blood flow, larvae were reared and infected with *C*. *albicans* as described and then placed in petri dishes containing 0.1 mg/ml valproic acid in E3 media plus PTU or 2 μM terfenadine in E3 media plus PTU and corresponding vehicle controls (water and DMSO, respectively). Larvae were observed for reduced heartbeat and loss of blood flow in the trunk and tail by 24 hours post treatment (or 24 hpi).

### Histology

Larvae were euthanized after 42 hpi in tricaine overdose and shipped overnight in PBS. Larvae were then fixed in 4% PFA at 4°C overnight, washed in PBS, and placed in 30% sucrose (MP Biomedicals 821713) in PBS for overnight or until the samples sank. Larvae samples were embedded in Tissue-Tek O.C.T. compound (OCT; Sakura Finetek 4583) and were kept at -80°C for at least 10 mins before sectioning. Sagittal 20-micron sections were collected with a Leica cryostat (CM3050S) and the sections were stained with DAPI (1 mg/ml; 1:2000 dilution) and Alexa Fluor 568 Phalloidin (Invitrogen A12380; 1:500 dilution) for 30 mins– 1 hour at room temperature. Then the slides were washed with PBS and mounted for imaging. Slides were then shipped back and sections were imaged on the Olympus Fluoview 1000 confocal microscope. Images were processed and pseudocolored in ImageJ.

### Statistical analysis & full raw data

Statistical analysis was performed in Graphpad Prism versions 6, 9 and 10 (Graphpad software, Inc., La Jolla, CA). All analysis was completed with non-parametric tests (Kruskall-Wallis for ANOVA and Mann-Whitney for pairwise) unless noted otherwise. P-values are indicated throughout the manuscript as: * p≤0.05, ** p≤0.01, *** p≤0.001, **** p≤0.0001. n.s. = not significant. The full raw data used for producing graphs is available as S1 Data.

## Supporting information

S1 Fig Neither initial *Candida* inoculum nor overall fungal burden indicates whether neutrophils will be recruited to the yolk sac or if the infection will result in dissemination events. *Tg(mpx*:*EGFP)* larvae were infected with NRG1^OEX^ as described previously and immediately imaged on the confocal to count the starting inoculum. (A) Example images of larvae just infected (0 hpi; top) and at 40 hpi (bottom). Images demonstrate little difference in starting inoculum, despite different infection results. Dissemination of fish, without phagocyte recruitment and with recruitment, are shown, respectively, below in panels (i) and (ii), with asterisks indicating disseminated yeast. Scale bar = 150 μm. (B) CFUs of screened larvae at 0 hpi. Bars show median and interquartile range of yeast per fish. Pooled from three experiments, n = 18 infected fish, median colonies per plate is 19. (C) CFUs from each type of recruitment/dissemination score. Bars indicate median and interquartile range. Pooled from three experiments, left to right n = 10, 4, 3, 10. Stats: Kruskal-Wallis with Dunn’s post-test, no differences with p<0.5. (D) *Candida* burden as measured by the number of fluorescent pixels quantified from confocal Z-stack images as pooled from >3 independent experiments, left to right n = 59, 11, 13, 16 per time point.
(PDF)

S2 Fig Location of *C*. *albicans* does not affect likelihood of dissemination. *Tg(mpeg*:*GAL4)/(UAS*:*nfsb-mCherry)* fish were crossed with *Tg(fli1*:*EGFP)* fish and infected with NRG1^OEX^-iRFP as described. Images taken at 24, 30, and 40 hpi were used to quantify the pixel distance of fluorescent *Candida* away from the (A) edge of the sac outlined by the DIC image, or (B) GFP positive endothelial cells lining the vasculature around the yolk sac. (C-D) Dissemination is not associated with vicinity to the yolk edge or to blood vessels. Graphs represent the average distances, for each larva, of each pixel to the closest the yolk edge or EGFP+ cell. Stats: Mann-Whitney tests at each time point. There were no differences with p<0.05. (C-D) Dissemination is not associated with vicinity to the yolk edge or to blood vessels. (E-F) To represent the data differently, each fish analyzed in panels C & D was binned for distance to yolk edge or vasculature, in bins of 10 pixels, and the categories of fish in each bin were stacked, including ND fish and those with visible dissemination at 24 hpi, 30 hpi or 40 hpi. Pooled from 3 experiments, n = 26 non-disseminated larvae and 19 disseminated larvae at 40 hpi.
(PDF)

S3 Fig Killed yeast do not readily disseminate from the yolk sac or elicit immune cell response. *Tg(mpx*:*EGFP)* larvae were infected with a wild type *C*. *albicans* and kept at 21°C for the course of infection. *Candida* was UV killed and stained with AlexaFluor 555 prior to injection in the yolk. Larvae were followed for neutrophil recruitment and fungal dissemination at 24, 30, and 40 hpi. Care was taken to remove larvae with dissemination events that occurred before 24 hpi, to ensure later dissemination events were a result of normal infection processes rather than artifactual injection into the bloodstream. (A) Percent larvae with dissemination of UV inactivated or live fungi at 30 and 40 hpi. For level of dissemination scoring method see Fig 3. Pooled from 4 experiments, left to right n = 77, 85. Stats: Fisher’s exact test, *** p ≤ 0.001, **** p ≤ 0.0001. (B) Percent larvae with neutrophil recruitment to UV inactivated, heat killed, or live fungi. Same larvae as followed in panel A. Stats: Fisher’s exact test, **** p ≤ 0.0001.
(PDF)

S4 Fig Levels of *tnfa*:*EGFP* at the site of infection correlate with phagocyte recruitment but not dissemination. *Tg(lysC*:*Ds-Red)/Tg(tnfα*:*GFP)* larvae with red fluorescent neutrophils and green fluorescence with *tnfα* expression were infected with a yeast-locked *C*. *albicans* as described for Fig 4. Total GFP+ pixels at 40 hpi were quantified. This was pooled from 5 experiments but was underpowered. N = 7, 9 and 5 larvae from L to R. Stats: Kruskal-Wallis with multiple comparisons. p = 1.0 and 0.432 respectively for ND/NR vs. ND/R and D/R.
(PDF)

S5 Fig Overall decrease in the number of phagocytes at the site of infection after dissemination starts. *Tg(mpeg*:*GAL4/UAS*:*nfsb-mCherry)/Tg(mpx*:*EGFP)* larvae with green fluorescent neutrophils and red fluorescent macrophages were infected as described and scored at 24 hpi. (A-B) Images at 24, 30 and 40 hpi were quantified as to the number of fluorescent macrophages (A) and neutrophils (B) at the infection site at each time point. Stats: Kruskall-Wallis with Dunn’s post-test. * p<0.05, *** p<0.001. N = 12 larvae with original score of Recruited/Disseminated.
(PDF)

S6 Fig Clodronate liposomes and metronidazole treatment are effective methods for macrophage ablation. *Tg(mpeg*:*GAL4/UAS*:*nfsb-mCherry)/Tg(mpx*:*EGFP)* larvae with green fluorescent neutrophils and red fluorescent macrophages were used to check efficiency of ablation methods. (A-C) Larvae were either injected at 28 hpf with 8–10 nl of control or clodronate liposomes (3:1:1 lipsomes:phenol red:10 kDa dextran) in the caudal vein. (B-D) Larvae were bathed in 20 mM metronidazole or vehicle (E3 water) for 4 hours following infection and 10 mM metronidazole thereafter. (A) Images of control and clodronate liposome treated larva, scale bar = 150 μm. (B) Number of macrophages or neutrophils counted in a 6 somite region in the trunk at 40 hpi. Bars indicate the median and interquartile range. Liposome treated larvae pooled from 4 experiments, left to right, n = 8, 18, 8, and 18. (C) Fungal burden quantified from confocal Z-stack images from 4 experiments (n = 18 controls and 18 clodronate-treated). (D) Images of control and metronidazole treated larvae. (E) Number of macrophages or neutrophils counted in the same region as B. Bars indicate the median and interquartile range. Pooled from 4 experiments, left to right, n = 8, 30, 11, and 19. (F) Fungal burden quantified from confocal Z-stack images from 4 experiments (n = 39 controls and 18 metronidazole-treated). A one-way ANOVA with Kruskall-Wallis post-test was used to test groups in panels B & E and Mann-Whitney for panels C & F, * ≤ 0.05, ** p ≤ 0.01, *** p ≤ 0.001, **** p ≤ 0.0001, n.s. = not significant p>0.05.
(PDF)

S7 Fig Chemical macrophage ablation does not alter dissemination rates. *Tg(mpeg*:*GAL4/UAS*:*nfsb-mCherry)/Tg(mpx*:*EGFP)* larvae were also used to examine phagocyte recruitment to the infection site. (A) Percent larvae with dissemination in clodronate- (left) and metronidazole- (right) treated larvae. Fisher’s exact test based on numbers shown below in figure, n.s. p>0.05. Pooled from 4 experiments. (B) Infection progression was scored as in Fig 1, with fish grouped by initial score (top, 24 hpi) and then by final score (bottom, 40 hpi). Fish and experiment numbers are the same as in Panel A.
(PDF)

S8 Fig Ablation of macrophages by morpholino oligonucleotide does not alter dissemination frequencies. *Tg(mpeg*:*GAL4)/(UAS*:*nfsb-mCherry)/Tg(mpx*:*EGFP)* embryos were injected at the 1–2 cell stage with a combination of splice blocking and translational blocking *pu*.*1* morpholino oligonucleotides to inhibit macrophage development. A Cascade blue fluorescent 10 kDa dextran was injected with the morpholino mix, and larvae were screened for correct injection of the morpholino mix following infection with NRG1^OEX^-iRFP. (A-B) Percent larvae with dissemination at 40 hpi. Pooled from 3 independent experiments (n = 54 control larvae and n = 54 morphant larvae). Stats: Fisher’s exact test, n.s. p>0.05 (C) Representative images of control and ablated larvae at 40 hpi. Images of the anterior and posterior of each fish was stitched with ImageJ, red in yolk is background autofluorescence. Purple outlined boxes are blown up in panel D. White arrowhead points to intact macrophage in control fish. Scale bar = 150 μm. (D) Blow-ups of small regions of the head from stitched images in (C) to show loss of macrophages in tissue; the only red fluorescence is from pieces of macrophages (white arrowheads) that haven’t been cleared. Scale bar = 150 μm.
(PDF)

S9 Fig Macrophage ablation limits TNFα production without affecting overall burden. *Tg(LysC*:*Ds-Red)/Tg(tnfα*:*GFP)* larvae with red fluorescent neutrophils and green fluorescence with *tnfα* expression were treated with liposomes and infected with NRG1^OEX^-iRFP. (A) Correlation graph between recruited neutrophils and *tnfα* expression overlapping areas of NRG1^OEX^-iRFP fluorescence. (B) ImageJ was used to make masks of the fluorescent channel for the yeast. The number of neutrophil (dsRed) or *tnfα* (GFP) pixels in the area of yeast was measured from these masks in MATLAB. Data pooled from 5 experiments, total fish used for quantification, left to right: n = 10, 21, 9, 23. Violin plots. Stats: Mann-Whitney. * p ≤ 0.05. (C) *Candida* burden is not affected by clodronate-mediated macrophage ablation. Total number of *Candida* pixels at the infection site shown as medians and 95% CI. Stats: Mann-Whitney, all p>0.05, not significant.
(PDF)

S10 Fig Chemical inhibitors of heart beat reduce blood flow, but not phagocyte recruitment or overall yeast dissemination. *Tg(Mpeg*:*GAL4/UAS*:*nfsb-mCherry)/Tg(mpo*:*EGFP)* larvae were bathed with 2 μM terfenadine/DMSO vehicle or in 0.1 mg/ml valproic acid/E3 water vehicle following infection with NRG1^OEX^-iRFP. (A) Representative images of infected larvae at 40 hpi treated with control or chemical blood flow blockers. (B) Percent area of the infection site with recruited macrophages and neutrophils. Pooled from 3 experiments with terfenadine, left to right, n = 11, 9, 25, and 23. Pooled from 3 experiments with valproic acid, left to right, n = 9, 7, 26, and 15. Stats: two-way ANOVA and Sidak’s multiple comparison’s test, * ≤ 0.05. (C) Dissemination scores of terfenadine treated larvae (pooled from 3 independent experiments) and valproic acid treated larvae (pooled from 3 independent experiments). (D) Details of numbers of individual fish and statistical analysis of experiments shown in Panel C. Fisher’s exact test, n.s. not significant.
(PDF)

S11 Fig In the context of chemical blockade of blood flow, macrophage ablation reduces *tnfa* expression but doesn’t affect fungal burden. Rac2-D57N zebrafish were crossed to *Tg(tnfα*:*GFP)* for neutrophil deficient offspring. All larvae were injected as previously with clodronate liposomes for macrophage ablation, and control or terfenadine for blood flow blockade. Larvae were infected with NRG1^OEX^-iRFP. (A) Representative images of Rac2-D57Nx*Tg(tnfα*:*GFP)* larvae at 40 hpi. Fish were scored by eye for dissemination and confocal images were chosen as close as possible to the median scores for both *TNFa*:*GFP* fluorescence and fungal burden. Note that disseminated yeast are not included in the images, which are focused on the infection site *tnfa* expression. Scale bar = 100 μm. (B) Very low levels of *tnfa* expression in all fish treated with clodronate is unaffected by blockade of blood flow or neutrophil activity. Quantification of *tnfα*:*GFP* positive pixels in the area of *Candida*, pooled from 5 experiments, left to right n = 13, 25, 12, 23, 13, 24, 12, and 21. Stats: Kruskal-Wallis with Dunn’s post-test, n.s. (C) Total fungal burden as quantified by the number of fluorescent *Candida* pixels in the whole fish (yolk plus body). Pooled from 6 experiments, left to right n = 15, 21, 8, and 10. Stats: Kruskal Wallis with Dunn’s post-test, n.s. Same fish quantified in panels B and C.
(PDF)

S12 Fig Outline of MATLAB workflows. This document outlines the steps taken in the MATLAB workflows used for image quantification, referencing the MATLAB scripts that are included as Supporting Information.
(PDF)

S1 Script New_TNF_express. Quantifies the number of TNFα or neutrophil pixels in the area of *C*. *albicans* pixels (Figs 4 and 7, S9 Fig and S11 Fig). It also measures the number of fluorescent pixels in the image, so *C*. *albicans* growth can be measured over time. Note that these calculations were made for individual confocal z-slices and not from maximum projections.
(MLX_CORR)

S2 Script Allison_candida_amounts_used. Measures the shortest distance between each pixel and the yolk edge, for each disseminated pixel, as shown for Fig 6D–6F.
(MLX_CORR)

S3 Script New_real_Bins_used. Bins data from “Allison_candida_amounts_used” by 1-pixel increments and compiles them into a single file. Note that these calculations were made for individual confocal z-slices and not from maximum projections.
(MLX_CORR)

S4 Script Allison_combining_candida_amounts_used. Uses data from the script “Allison_candida_amounts_used” to quantify the total level of burden in the yolk and the total amount of yeast that were disseminated.
(MLX_CORR)

S1 Movie As described in Fig 2A. *Tg(mpeg1*:*GAL4/UAS*:*nfsb-mCherry)/Tg(mpx*:*EGFP)* larvae were infected with yeast-locked *C*. *albicans* and scored for recruitment of phagocytes and dissemination of yeast at 24 hpi. A time-lapse of a larva with recruitment was taken over the course of 7 hours starting at ~32 hpi. Neutrophils and macrophages were observed moving within and away from the infection site carrying yeast.
(MOV)

S2 Movie As described in Fig 2A, but from an independent experiment. *Tg(mpeg1*:*GAL4/UAS*:*nfsb-mCherry)/Tg(mpx*:*EGFP)* larvae were infected with yeast-locked *C*. *albicans* and scored for recruitment of phagocytes and dissemination of yeast at 24 hpi. A time-lapse of fish with recruitment was taken over the course of 7 hours starting at ~32 hpi. Neutrophils and macrophages were observed moving within and away from the infection site carrying yeast.
(MOV)

S3 Movie As described in Fig 2C. *Tg(mpeg1*:*GAL4)x(UAS*:*Kaede)* larvae were infected with yeast-locked *C*. *albicans* and scored for recruitment of macrophages and dissemination of yeast at 24 hpi. Macrophages near the infection site of larvae with immune recruitment were photo-switched at 24 hpi. A time-lapse movie was taken over the course of 7 hours starting at ~46 hpi. A photo-converted macrophage moves out of the blood stream into tail tissue.
(MOV)

S4 Movie As described in Fig 2D. *Tg(mpeg1*:*GAL4)x(UAS*:*Kaede)* larvae were infected with yeast-locked *C*. *albicans* and scored for recruitment of macrophages and dissemination of yeast at 24 hpi. Macrophages near the infection site of larvae with immune recruitment were photo-switched at 24 hpi. A time-lapse movie taken over the course of 7 hours starting at ~32 hpi. Intracellular yeast growth and apparent NLE events are highlighted.
(MOV)

S5 Movie *Tg(mpx:EGFP)* larvae were infected with hypofilamentous *C*. *albicans* strain *cph1*Δ/Δ*efg1*Δ/Δ-dTomato in the yolk as described and imaged at 44 hpi by time-lapse microscopy. Neutrophils (green) interacting with yeast (red) as they move down the tail tissue inside a blood vessel.
(MOV)

S6 Movie As described in Fig 4D. *Tg(tnfα*:*EGFP)* larvae with green fluorescence with *tnfα* expression were given control liposomes and infected with a yeast-locked *C*. *albicans* as described. Z series demonstrating typical *tnfα* expression at the infection site in a wild type larva.
(MOV)

S7 Movie As described in Fig 4D. *Tg(tnfα*:*EGFP)* larvae with green fluorescence with *tnfα* expression were given clodronate liposomes and infected with a yeast-locked *C*. *albicans* as described. Z series demonstrating background *tnfα* expression in a wild type larva.
(MOV)

S8 Movie As described in Fig 4E. Rac2-D57N/*Tg(tnfα*:*EGFP)* larvae with red fluorescent neutrophils and green fluorescence with *tnfα* expression were given control liposomes and infected with a yeast-locked *C*. *albicans* as described. Z series demonstrating typical *tnfα* expression at the infection site in a Rac2/D57N larva.
(MOV)

S9 Movie As described in Fig 4E. Rac2-D57N/*Tg(tnfα*:*EGFP)* larvae with red fluorescent neutrophils and green fluorescence with *tnfα* expression were given clodronate liposomes and infected with a yeast-locked *C*. *albicans* as described. Z series demonstrating background *tnfα* expression in a Rac2/D57N larva.
(MOV)

S10 Movie As described in Fig 4. Rac2-D57N/*Tg(tnfα*:*EGFP)* larvae with red fluorescent neutrophils and green fluorescence with *tnfα* expression were given control liposomes and infected with a yeast-locked *C*. *albicans* as described. Time-lapse movie taken over the course of 7 hours starting at ~32 hpi. Macrophage-like cells turn on *tnfa* expression as they move closer to the site of infection.
(MOV)

S11 Movie As described in Fig 4. Rac2-D57N/*Tg(tnfα*:*EGFP)* larvae with red fluorescent neutrophils and green fluorescence with *tnfα* expression were given clodronate liposomes and infected with a yeast-locked *C*. *albicans* as described. Time-lapse movie taken over the course of 7 hours starting at ~32 hpi. There is an absence of phagocytes turning on *tnfa* expression.
(MOV)

S12 Movie As described in S10 Fig. Tg*(mpeg*:*GAL4/UAS*:*nfsb-mCherry)/Tg(mp*x:*EGFP)* larvae were bathed with 2 μM terfenadine/DMSO vehicle following infection with NRG1^OEX^-iRFP. Representative movie of infected larvae at ~32 hpi treated with 2 μM terfenadine, which blocks blood flow.
(MOV)

S13 Movie As described in S10 Fig. *Tg(mpeg*:*GAL4/UAS*:*nfsb-mCherry)/Tg*(*mp*x:*EGFP)* larvae were bathed in 0.1 mg/ml valproic acid/E3 water vehicle following infection with NRG1^OEX^-iRFP. Representative movie of infected larvae at ~32 hpi treated with 0.1 mg/ml valproic acid, which blocks blood flow.
(MOV)

S14 Movie As described in Fig 5G. *Tg(fli1*:*EGFP)* larvae, with GFP-expressing endothelial cells, were infected with *NRG1*^OEX^-iRFP as previously described. A vehicle-treated *Tg(fli1*:*EGFP)* fish with intact blood flow was imaged at ~32 hpi by time-lapse on the confocal.
(MOV)

S15 Movie As described in Fig 5G. *Tg(fli1*:*EGFP)* larvae, with GFP-expressing endothelial cells, were infected with *NRG1*^OEX^-iRFP as previously described. A vehicle-treated *Tg(fli1*:*EGFP)* fish with intact blood flow was imaged at ~32 hpi by time-lapse on the confocal.
(MOV)

S16 Movie As described in Fig 7. RAC2-D57N/*Tg(tnfα*:*GFP)* larvae were injected with control liposomes and infected with the wild type CAF2-iRFP *C*. *albicans* as described for the yeast-locked infections. Fish were imaged by confocal microscopy between 32 and 40 hpi.
(MOV)

S17 Movie As described in Fig 7. RAC2-D57N/*Tg(tnfα*:*GFP)* larvae were injected with clodronate liposomes and infected with the wild type CAF2-iRFP *C*. *albicans* as described for the yeast-locked infections. Fish were imaged by confocal microscopy between 32 and 40 hpi.
(MOV)

S18 Movie As described in Fig 7. *Tg(mpeg*:*GAL4/UAS*:*nfsb-mCherry)/Tg*(*mp*x:*EGFP)* larvae were infected with the wild type Caf2-iRFP *C*. *albicans* as described for the yeast-locked infections. Fish were imaged by confocal microscopy between 32 and 40 hpi.
(MOV)

S1 Data Excel spreadsheet with data used to produce all graphs in Figs 1–7. (XLSX)
