# Supplementary figures and images for: Redundant Trojan horse and endothelial-circulatory mechanisms for host-mediated spread of Candida albicans yeast
Source: PLoS Pathog. 2020 Aug 10;16(8):e1008414. doi: 10.1371/journal.ppat.1008414 (PMC7447064; doi:10.1371/journal.ppat.1008414)

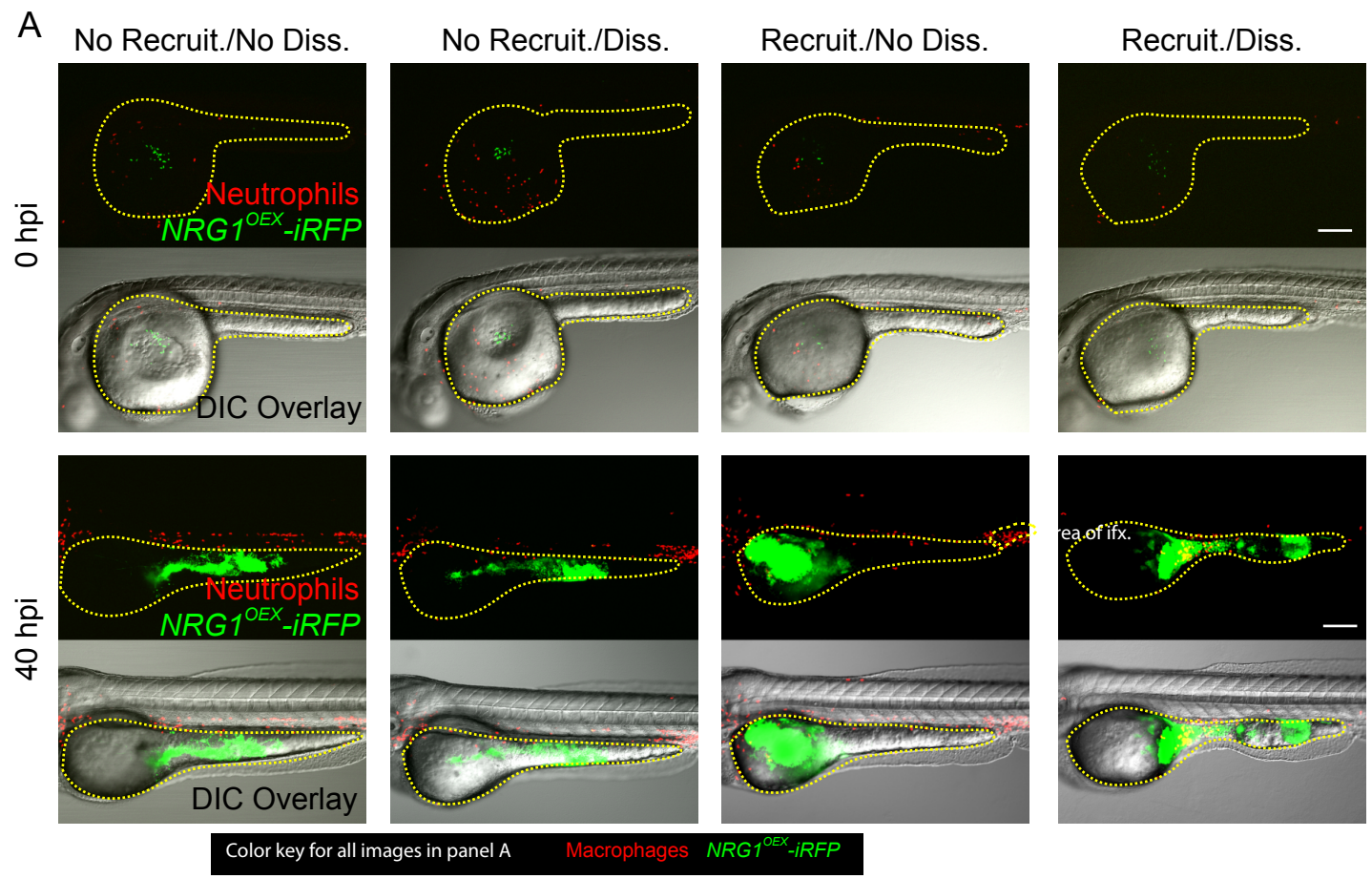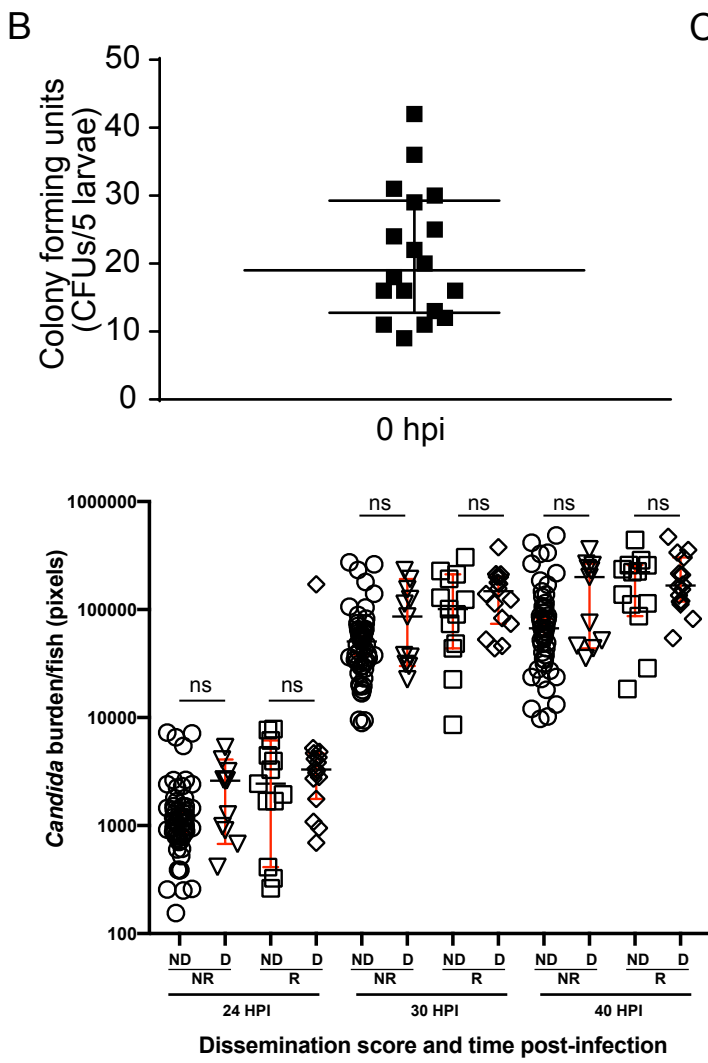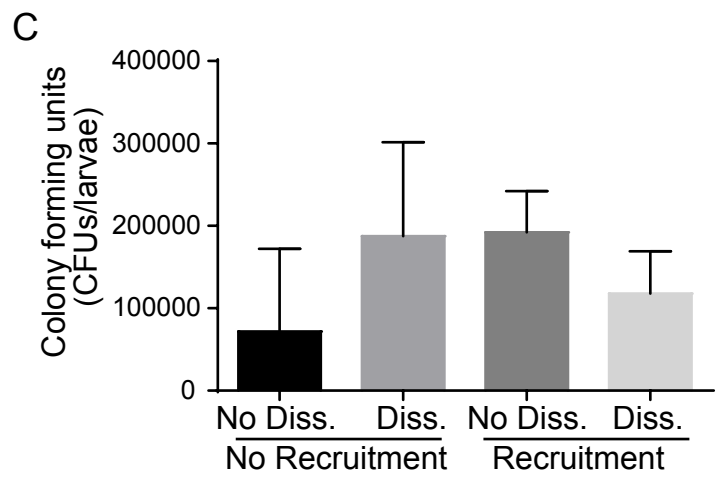

Supplement: S1 Fig — Tg(mpx:EGFP) larvae were infected with NRG1OEX as described previously and immediately imaged on the confocal to count the starting inoculum. (A) Example images of larvae just infected (0 hpi; top) and at 40 hpi (bottom). Images demonstrate little difference in starting inoculum, despite different infection results. Dissemination of fish, without phagocyte recruitment and with recruitment, are shown, respectively, below in panels (i) and (ii), with asterisks indicating disseminated yeast. Scale bar = 150 μm. (B) CFUs of screened larvae at 0 hpi. Bars show median and interquartile range of yeast per fish. Pooled from three experiments, n = 18 infected fish, median colonies per plate is 19. (C) CFUs from each type of recruitment/dissemination score. Bars indicate median and interquartile range. Pooled from three experiments, left to right n = 10, 4, 3, 10. Stats: Kruskal-Wallis with Dunn’s post-test, no differences with p<0.5. (D) Candida burden as measured by the number of fluorescent pixels quantified from confocal Z-stack images as pooled from >3 independent experiments, left to right n = 59, 11, 13, 16 per time point. (PDF) [file ppat.1008414.s001.pdf]

A

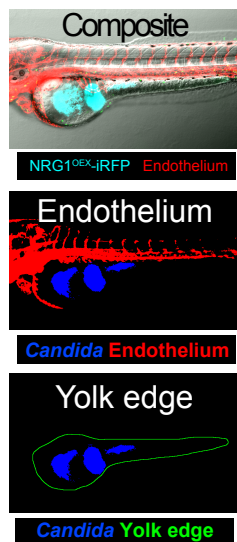

B

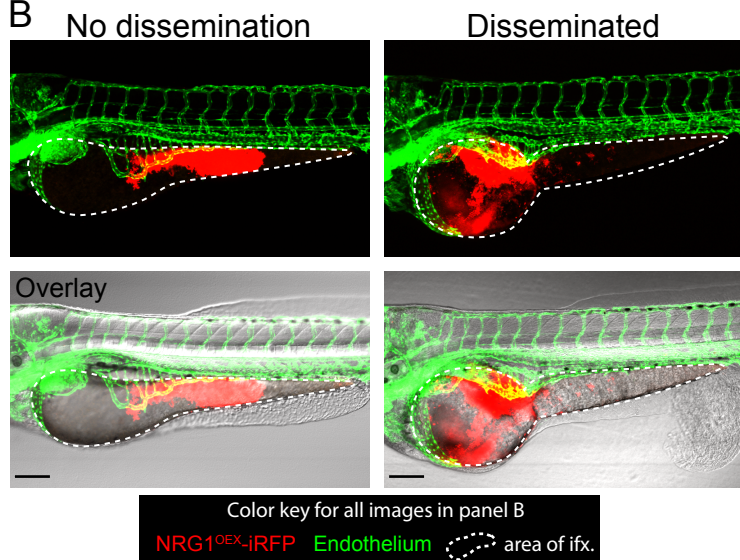

C

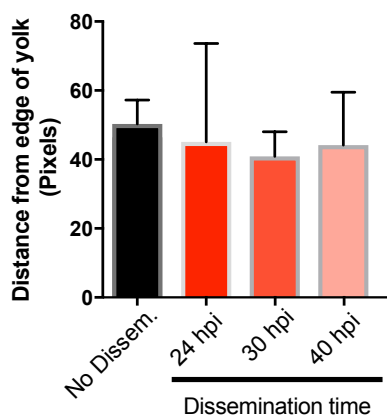

D

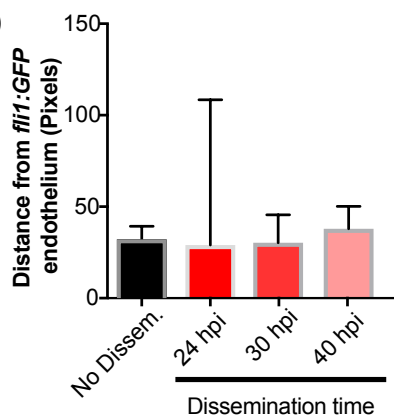

E

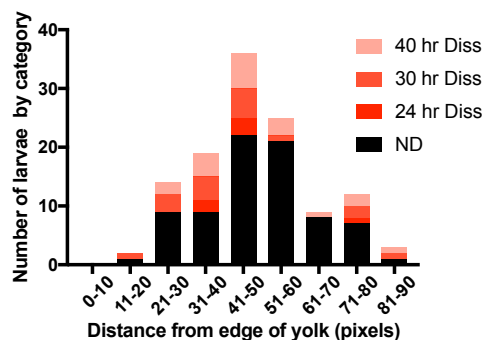

F

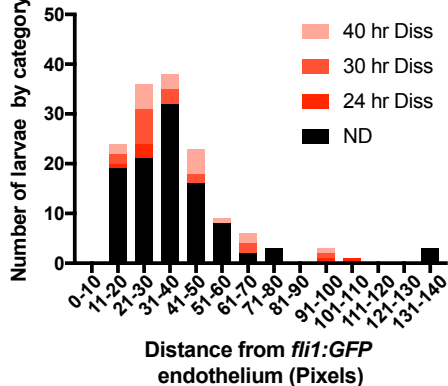

Supplement: S2 Fig — Tg(mpeg:GAL4)/(UAS:nfsb-mCherry) fish were crossed with Tg(fli1:EGFP) fish and infected with NRG1OEX-iRFP as described. Images taken at 24, 30, and 40 hpi were used to quantify the pixel distance of fluorescent Candida away from the (A) edge of the sac outlined by the DIC image, or (B) GFP positive endothelial cells lining the vasculature around the yolk sac. (C-D) Dissemination is not associated with vicinity to the yolk edge or to blood vessels. Graphs represent the average distances, for each larva, of each pixel to the closest the yolk edge or EGFP+ cell. Stats: Mann-Whitney tests at each time point. There were no differences with p<0.05. (C-D) Dissemination is not associated with vicinity to the yolk edge or to blood vessels. (E-F) To represent the data differently, each fish analyzed in panels C & D was binned for distance to yolk edge or vasculature, in bins of 10 pixels, and the categories of fish in each bin were stacked, including ND fish and those with visible dissemination at 24 hpi, 30 hpi or 40 hpi. Pooled from 3 experiments, n = 26 non-disseminated larvae and 19 disseminated larvae at 40 hpi. (PDF) [file ppat.1008414.s002.pdf]

A

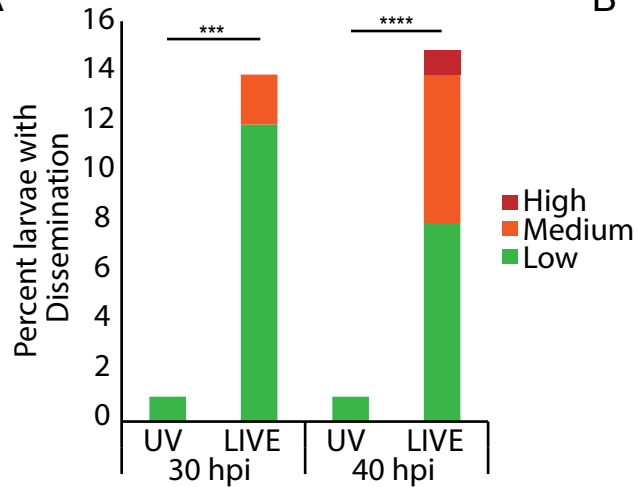

B

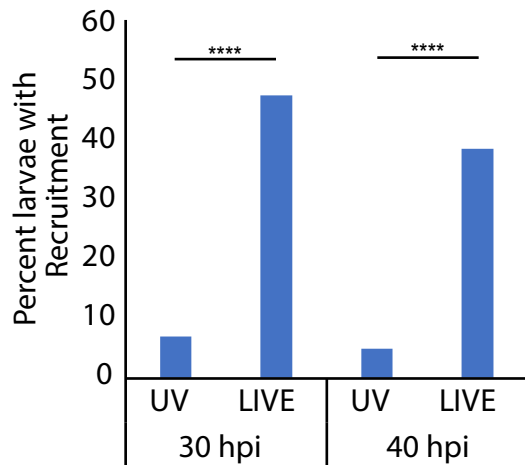

Fig. S3

Supplement: S3 Fig — Tg(mpx:EGFP) larvae were infected with a wild type C. albicans and kept at 21°C for the course of infection. Candida was UV killed and stained with AlexaFluor 555 prior to injection in the yolk. Larvae were followed for neutrophil recruitment and fungal dissemination at 24, 30, and 40 hpi. Care was taken to remove larvae with dissemination events that occurred before 24 hpi, to ensure later dissemination events were a result of normal infection processes rather than artifactual injection into the bloodstream. (A) Percent larvae with dissemination of UV inactivated or live fungi at 30 and 40 hpi. For level of dissemination scoring method see Fig 3. Pooled from 4 experiments, left to right n = 77, 85. Stats: Fisher’s exact test, *** p ≤ 0.001, **** p ≤ 0.0001. (B) Percent larvae with neutrophil recruitment to UV inactivated, heat killed, or live fungi. Same larvae as followed in panel A. Stats: Fisher’s exact test, **** p ≤ 0.0001. (PDF) [file ppat.1008414.s003.pdf]

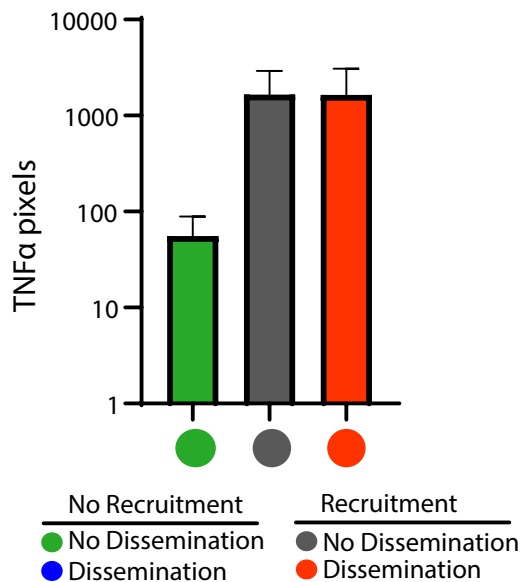

Fig. S4

Supplement: S4 Fig — Tg(lysC:Ds-Red)/Tg(tnfα:GFP) larvae with red fluorescent neutrophils and green fluorescence with tnfα expression were infected with a yeast-locked C. albicans as described for Fig 4. Total GFP+ pixels at 40 hpi were quantified. This was pooled from 5 experiments but was underpowered. N = 7, 9 and 5 larvae from L to R. Stats: Kruskal-Wallis with multiple comparisons. p = 1.0 and 0.432 respectively for ND/NR vs. ND/R and D/R. (PDF) [file ppat.1008414.s004.pdf]

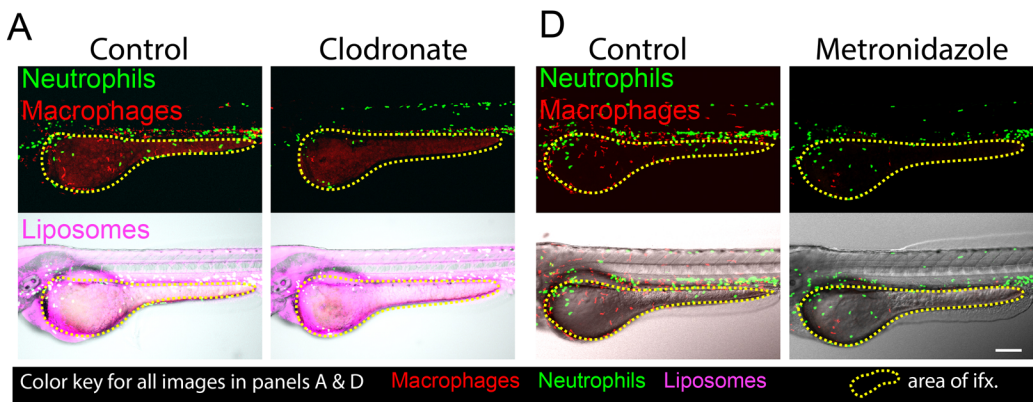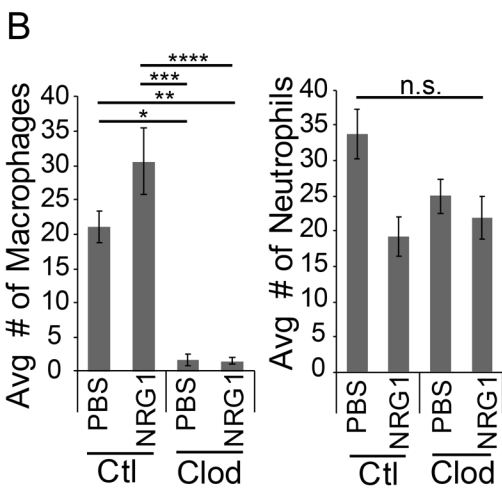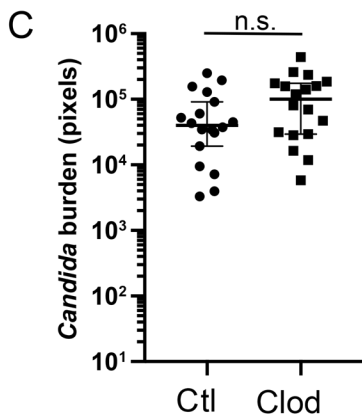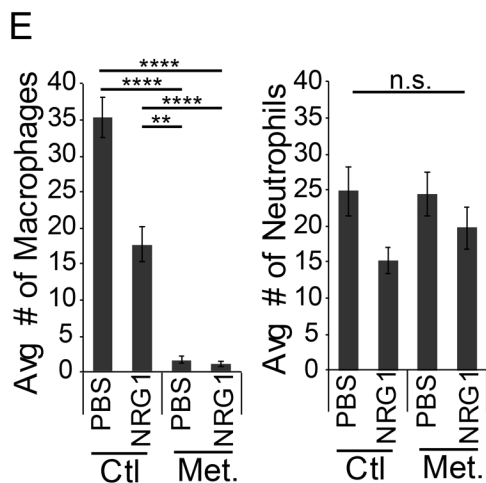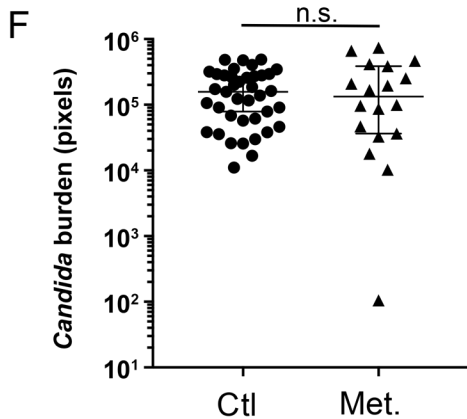

Supplement: S6 Fig — Tg(mpeg:GAL4/UAS:nfsb-mCherry)/Tg(mpx:EGFP) larvae with green fluorescent neutrophils and red fluorescent macrophages were used to check efficiency of ablation methods. (A-C) Larvae were either injected at 28 hpf with 8–10 nl of control or clodronate liposomes (3:1:1 lipsomes:phenol red:10 kDa dextran) in the caudal vein. (B-D) Larvae were bathed in 20 mM metronidazole or vehicle (E3 water) for 4 hours following infection and 10 mM metronidazole thereafter. (A) Images of control and clodronate liposome treated larva, scale bar = 150 μm. (B) Number of macrophages or neutrophils counted in a 6 somite region in the trunk at 40 hpi. Bars indicate the median and interquartile range. Liposome treated larvae pooled from 4 experiments, left to right, n = 8, 18, 8, and 18. (C) Fungal burden quantified from confocal Z-stack images from 4 experiments (n = 18 controls and 18 clodronate-treated). (D) Images of control and metronidazole treated larvae. (E) Number of macrophages or neutrophils counted in the same region as B. Bars indicate the median and interquartile range. Pooled from 4 experiments, left to right, n = 8, 30, 11, and 19. (F) Fungal burden quantified from confocal Z-stack images from 4 experiments (n = 39 controls and 18 metronidazole-treated). A one-way ANOVA with Kruskall-Wallis post-test was used to test groups in panels B & E and Mann-Whitney for panels C & F, * ≤ 0.05, ** p ≤ 0.01, *** p ≤ 0.001, **** p ≤ 0.0001, n.s. = not significant p>0.05. (PDF) [file ppat.1008414.s006.pdf]

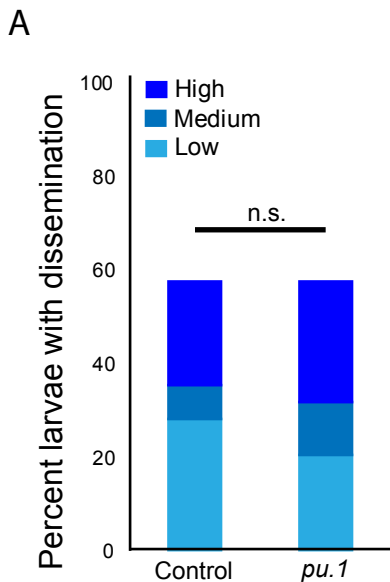

**B**

$p = 1.0000$

|                  | Control | <i>pu.1</i> |
|------------------|---------|-------------|
| NO DISSEMINATION | 16      | 16          |
| DISSEMINATION    | 31      | 31          |

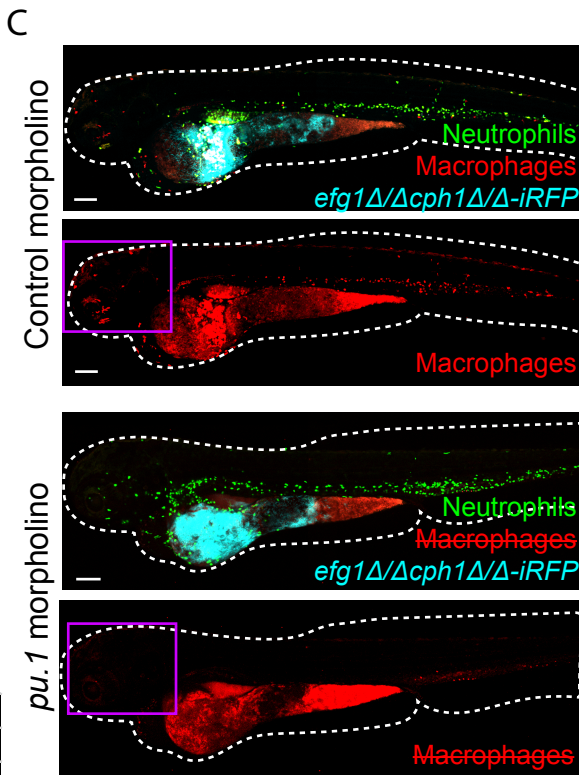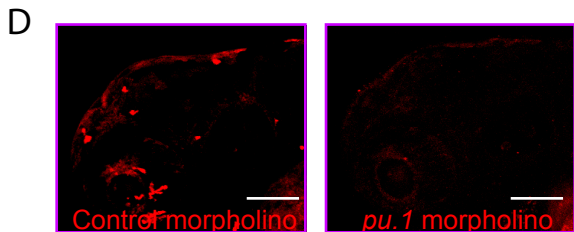

Fig. S8

Supplement: S8 Fig — Tg(mpeg:GAL4)/(UAS:nfsb-mCherry)/Tg(mpx:EGFP) embryos were injected at the 1–2 cell stage with a combination of splice blocking and translational blocking pu.1 morpholino oligonucleotides to inhibit macrophage development. A Cascade blue fluorescent 10 kDa dextran was injected with the morpholino mix, and larvae were screened for correct injection of the morpholino mix following infection with NRG1OEX-iRFP. (A-B) Percent larvae with dissemination at 40 hpi. Pooled from 3 independent experiments (n = 54 control larvae and n = 54 morphant larvae). Stats: Fisher’s exact test, n.s. p>0.05 (C) Representative images of control and ablated larvae at 40 hpi. Images of the anterior and posterior of each fish was stitched with ImageJ, red in yolk is background autofluorescence. Purple outlined boxes are blown up in panel D. White arrowhead points to intact macrophage in control fish. Scale bar = 150 μm. (D) Blow-ups of small regions of the head from stitched images in (C) to show loss of macrophages in tissue; the only red fluorescence is from pieces of macrophages (white arrowheads) that haven’t been cleared. Scale bar = 150 μm. (PDF) [file ppat.1008414.s008.pdf]

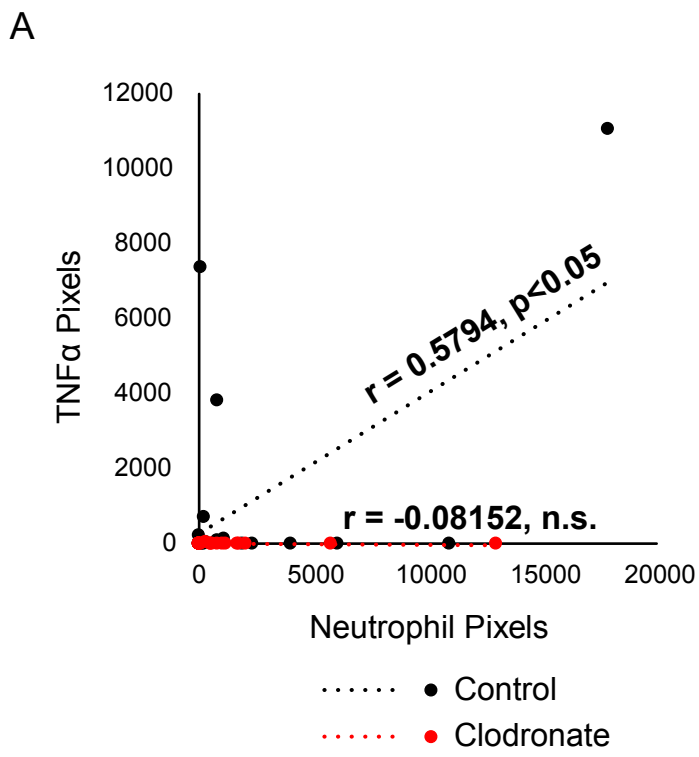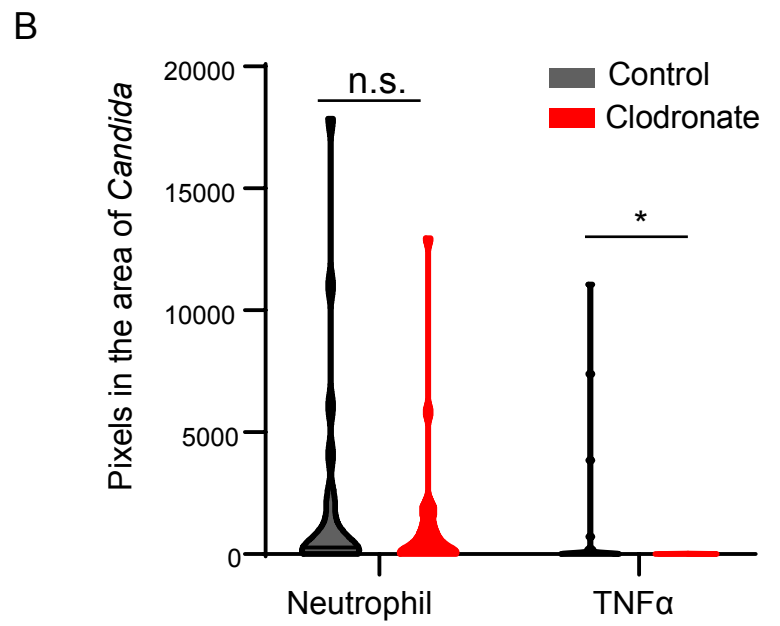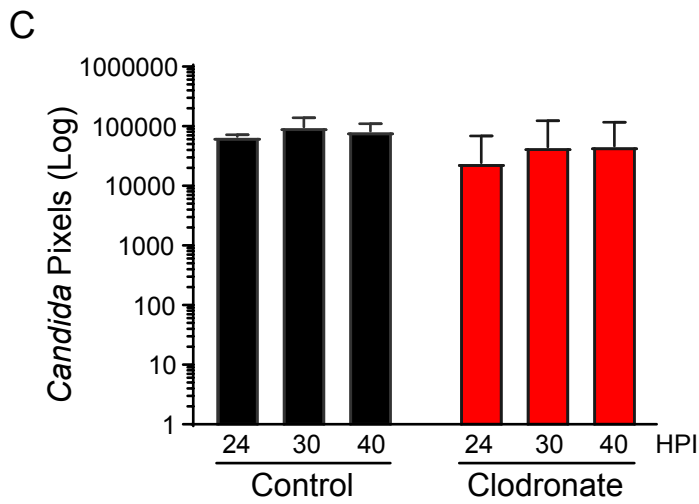

Fig. S9

Supplement: S9 Fig — Tg(LysC:Ds-Red)/Tg(tnfα:GFP) larvae with red fluorescent neutrophils and green fluorescence with tnfα expression were treated with liposomes and infected with NRG1OEX-iRFP. (A) Correlation graph between recruited neutrophils and tnfα expression overlapping areas of NRG1OEX-iRFP fluorescence. (B) ImageJ was used to make masks of the fluorescent channel for the yeast. The number of neutrophil (dsRed) or tnfα (GFP) pixels in the area of yeast was measured from these masks in MATLAB. Data pooled from 5 experiments, total fish used for quantification, left to right: n = 10, 21, 9, 23. Violin plots. Stats: Mann-Whitney. * p ≤ 0.05. (C) Candida burden is not affected by clodronate-mediated macrophage ablation. Total number of Candida pixels at the infection site shown as medians and 95% CI. Stats: Mann-Whitney, all p>0.05, not significant. (PDF) [file ppat.1008414.s009.pdf]

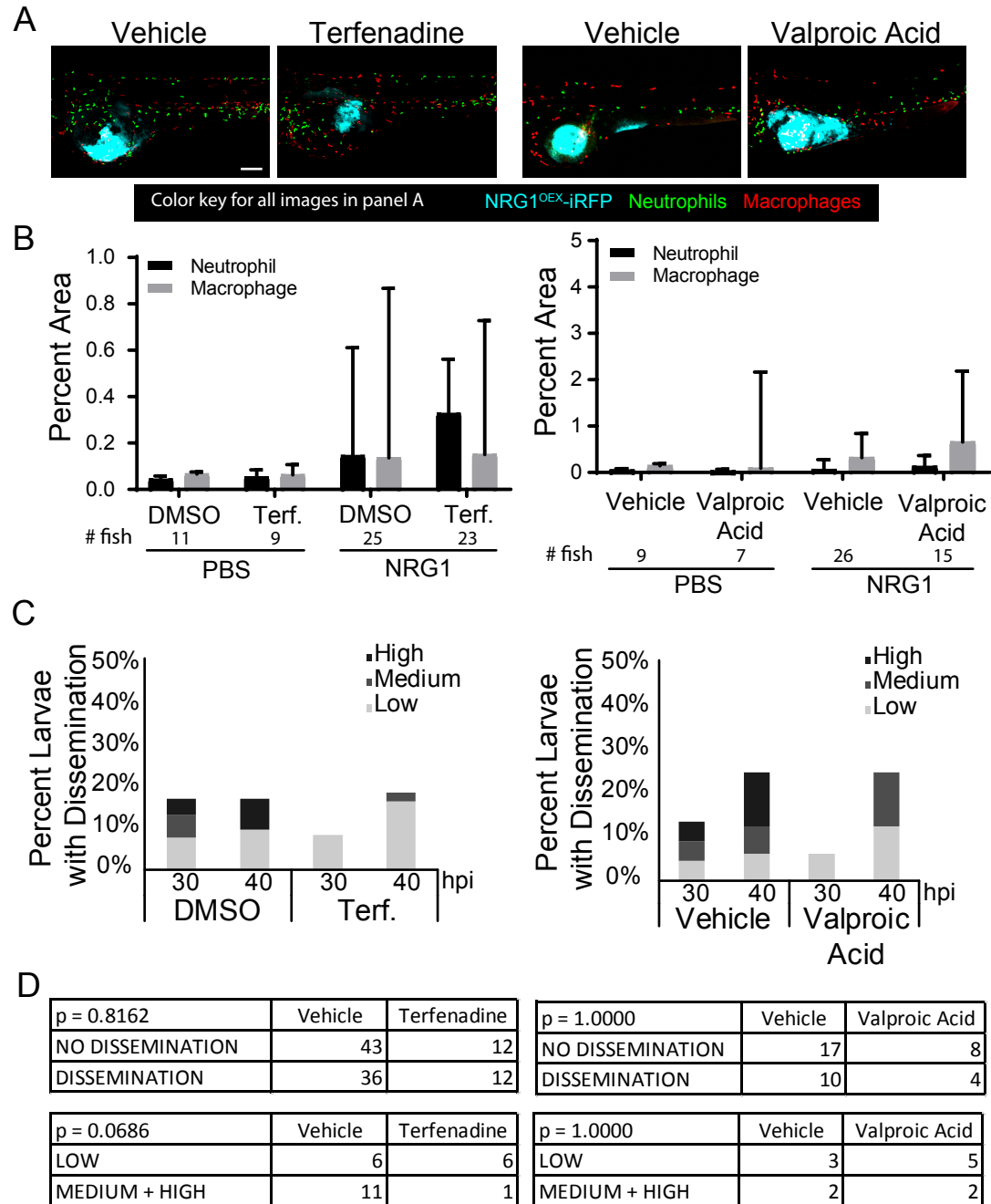

Fig. S10

Supplement: S10 Fig — Tg(Mpeg:GAL4/UAS:nfsb-mCherry)/Tg(mpo:EGFP) larvae were bathed with 2 μM terfenadine/DMSO vehicle or in 0.1 mg/ml valproic acid/E3 water vehicle following infection with NRG1OEX-iRFP. (A) Representative images of infected larvae at 40 hpi treated with control or chemical blood flow blockers. (B) Percent area of the infection site with recruited macrophages and neutrophils. Pooled from 3 experiments with terfenadine, left to right, n = 11, 9, 25, and 23. Pooled from 3 experiments with valproic acid, left to right, n = 9, 7, 26, and 15. Stats: two-way ANOVA and Sidak’s multiple comparison’s test, * ≤ 0.05. (C) Dissemination scores of terfenadine treated larvae (pooled from 3 independent experiments) and valproic acid treated larvae (pooled from 3 independent experiments). (D) Details of numbers of individual fish and statistical analysis of experiments shown in Panel C. Fisher’s exact test, n.s. not significant. (PDF) [file ppat.1008414.s010.pdf]

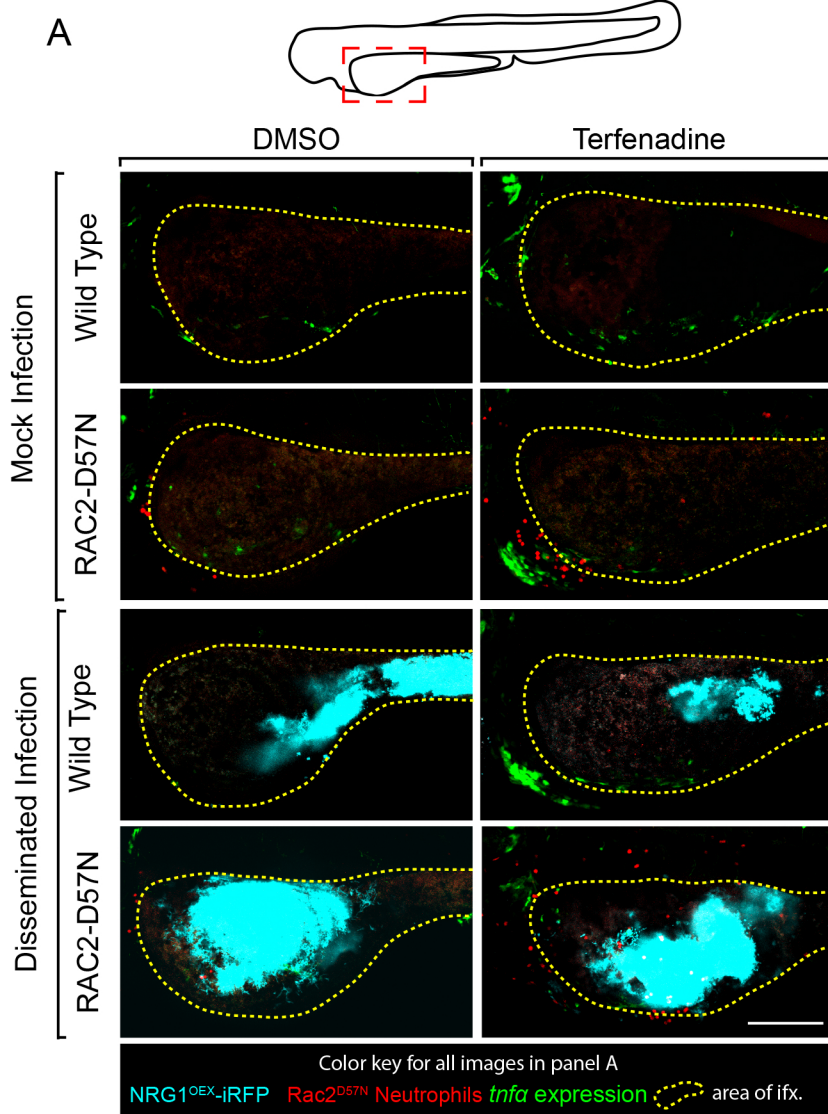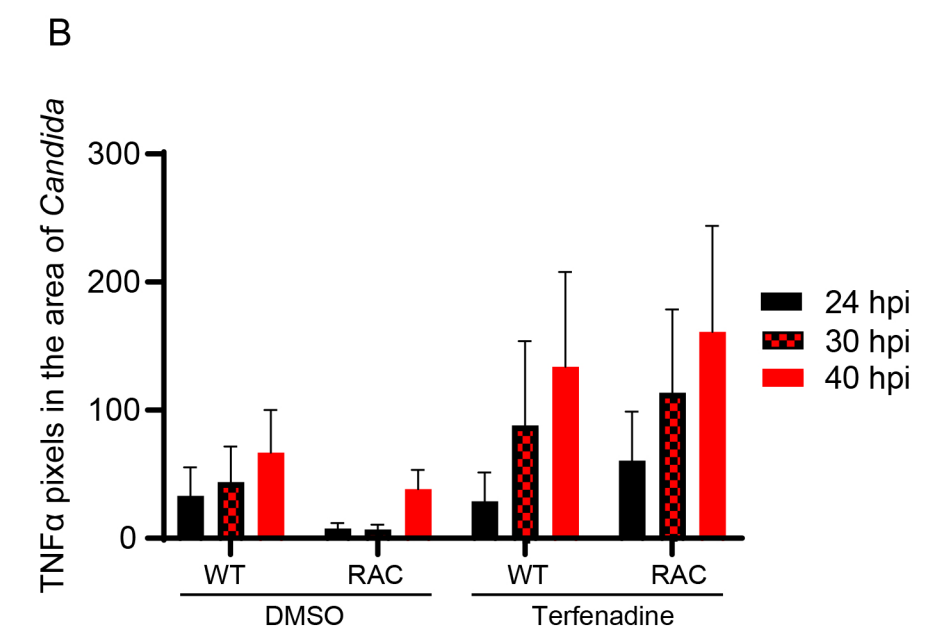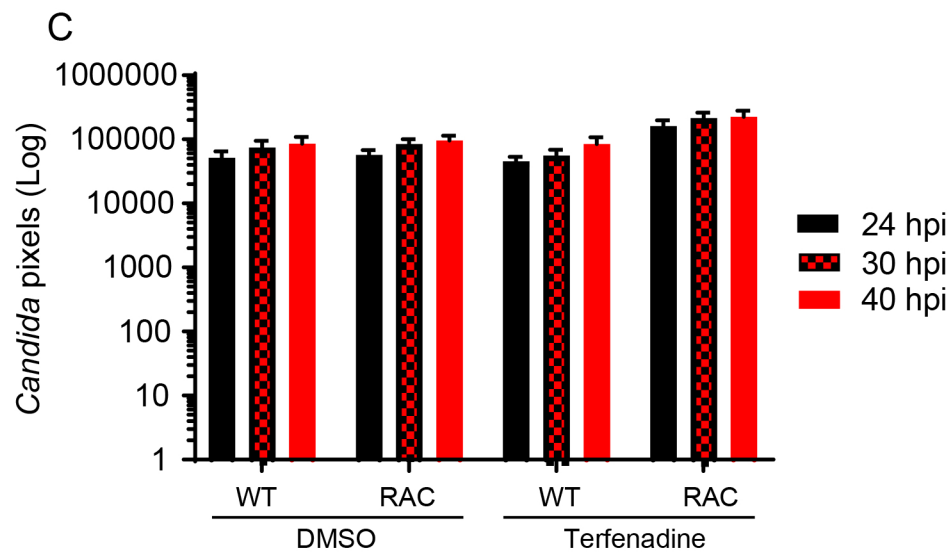

Supplement: S11 Fig — Rac2-D57N zebrafish were crossed to Tg(tnfα:GFP) for neutrophil deficient offspring. All larvae were injected as previously with clodronate liposomes for macrophage ablation, and control or terfenadine for blood flow blockade. Larvae were infected with NRG1OEX-iRFP. (A) Representative images of Rac2-D57NxTg(tnfα:GFP) larvae at 40 hpi. Fish were scored by eye for dissemination and confocal images were chosen as close as possible to the median scores for both TNFa:GFP fluorescence and fungal burden. Note that disseminated yeast are not included in the images, which are focused on the infection site tnfa expression. Scale bar = 100 μm. (B) Very low levels of tnfa expression in all fish treated with clodronate is unaffected by blockade of blood flow or neutrophil activity. Quantification of tnfα:GFP positive pixels in the area of Candida, pooled from 5 experiments, left to right n = 13, 25, 12, 23, 13, 24, 12, and 21. Stats: Kruskal-Wallis with Dunn’s post-test, n.s. (C) Total fungal burden as quantified by the number of fluorescent Candida pixels in the whole fish (yolk plus body). Pooled from 6 experiments, left to right n = 15, 21, 8, and 10. Stats: Kruskal Wallis with Dunn’s post-test, n.s. Same fish quantified in panels B and C. (PDF) [file ppat.1008414.s011.pdf]
