# Supplementary material for: Redundant Trojan horse and endothelial-circulatory mechanisms for host-mediated spread of Candida albicans yeast
Source: PLoS Pathog. 2020 Aug 10;16(8):e1008414. doi: 10.1371/journal.ppat.1008414 (PMC7447064; doi:10.1371/journal.ppat.1008414)
Supplement: S5 Fig — Tg(mpeg:GAL4/UAS:nfsb-mCherry)/Tg(mpx:EGFP) larvae with green fluorescent neutrophils and red fluorescent macrophages were infected as described and scored at 24 hpi. (A-B) Images at 24, 30 and 40 hpi were quantified as to the number of fluorescent macrophages (A) and neutrophils (B) at the infection site at each time point. Stats: Kruskall-Wallis with Dunn’s post-test. * p<0.05, *** p<0.001. N = 12 larvae with original score of Recruited/Disseminated. (PDF) [file ppat.1008414.s005.pdf]

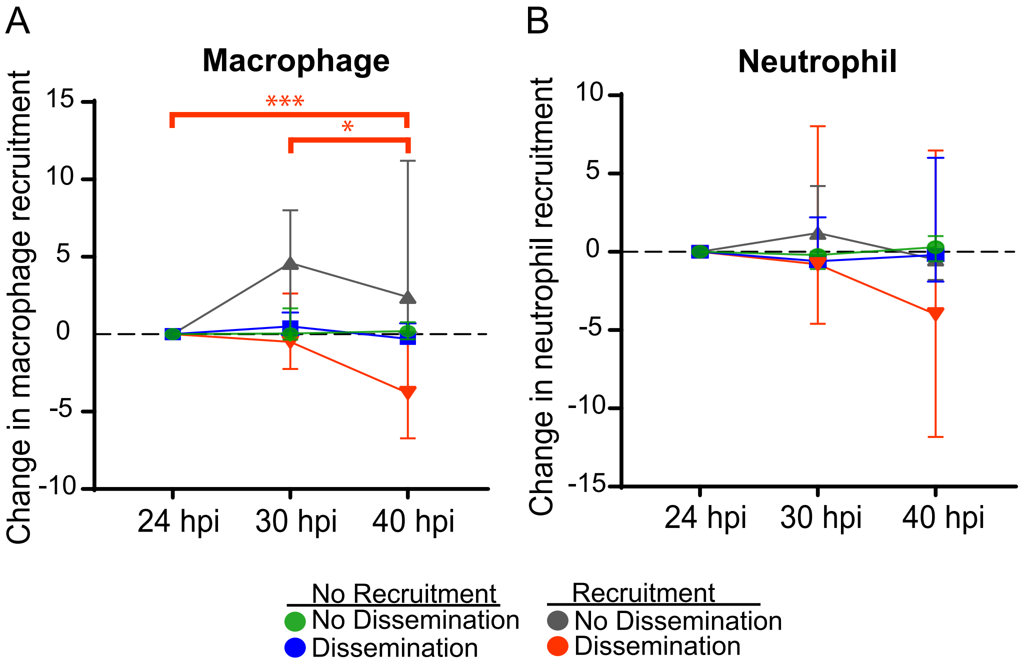
A
Macrophage
Change in macrophage recruitment
15
10
5
0
-5
-10
***
*
24 hpi
30 hpi
40 hpi
B
Neutrophil
Change in neutrophil recruitment
10
5
0
-5
-10
-15
24 hpi
30 hpi
40 hpi
No Recruitment
No Dissemination
Dissemination
Recruitment
No Dissemination
Dissemination

Fig. S5
