# Supplementary material for: Redundant Trojan horse and endothelial-circulatory mechanisms for host-mediated spread of Candida albicans yeast
Source: PLoS Pathog. 2020 Aug 10;16(8):e1008414. doi: 10.1371/journal.ppat.1008414 (PMC7447064; doi:10.1371/journal.ppat.1008414)
Supplement: S7 Fig — Tg(mpeg:GAL4/UAS:nfsb-mCherry)/Tg(mpx:EGFP) larvae were also used to examine phagocyte recruitment to the infection site. (A) Percent larvae with dissemination in clodronate- (left) and metronidazole- (right) treated larvae. Fisher’s exact test based on numbers shown below in figure, n.s. p>0.05. Pooled from 4 experiments. (B) Infection progression was scored as in Fig 1, with fish grouped by initial score (top, 24 hpi) and then by final score (bottom, 40 hpi). Fish and experiment numbers are the same as in Panel A. (PDF) [file ppat.1008414.s007.pdf]

A

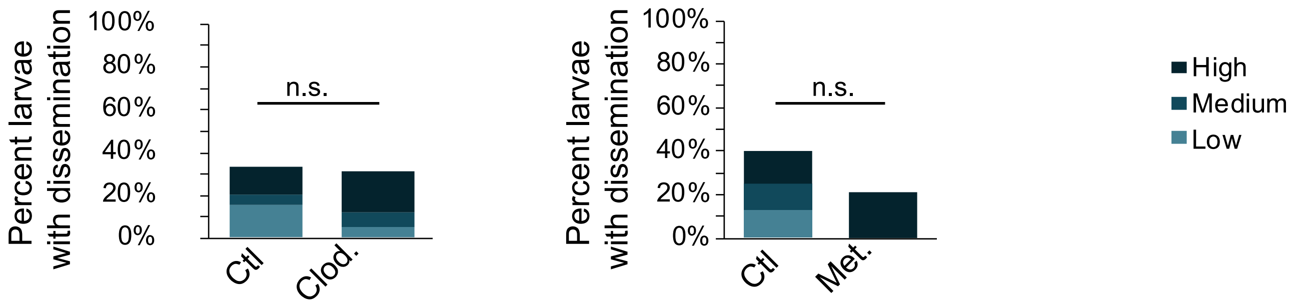

p = 1.0000	CONTROL	CLODRONATE
NO DISSEMINATION	40	36
DISSEMINATION	20	19

p = 0.5557	CONTROL	METRONIDAZOLE
NO DISSEMINATION	18	13
DISSEMINATION	13	6

p = 0.0824	CONTROL	CLODRONATE
LOW	9	3
MEDIUM + HIGH	11	16

p = 0.2554	CONTROL	METRONIDAZOLE
LOW	4	0
MEDIUM + HIGH	9	6

B

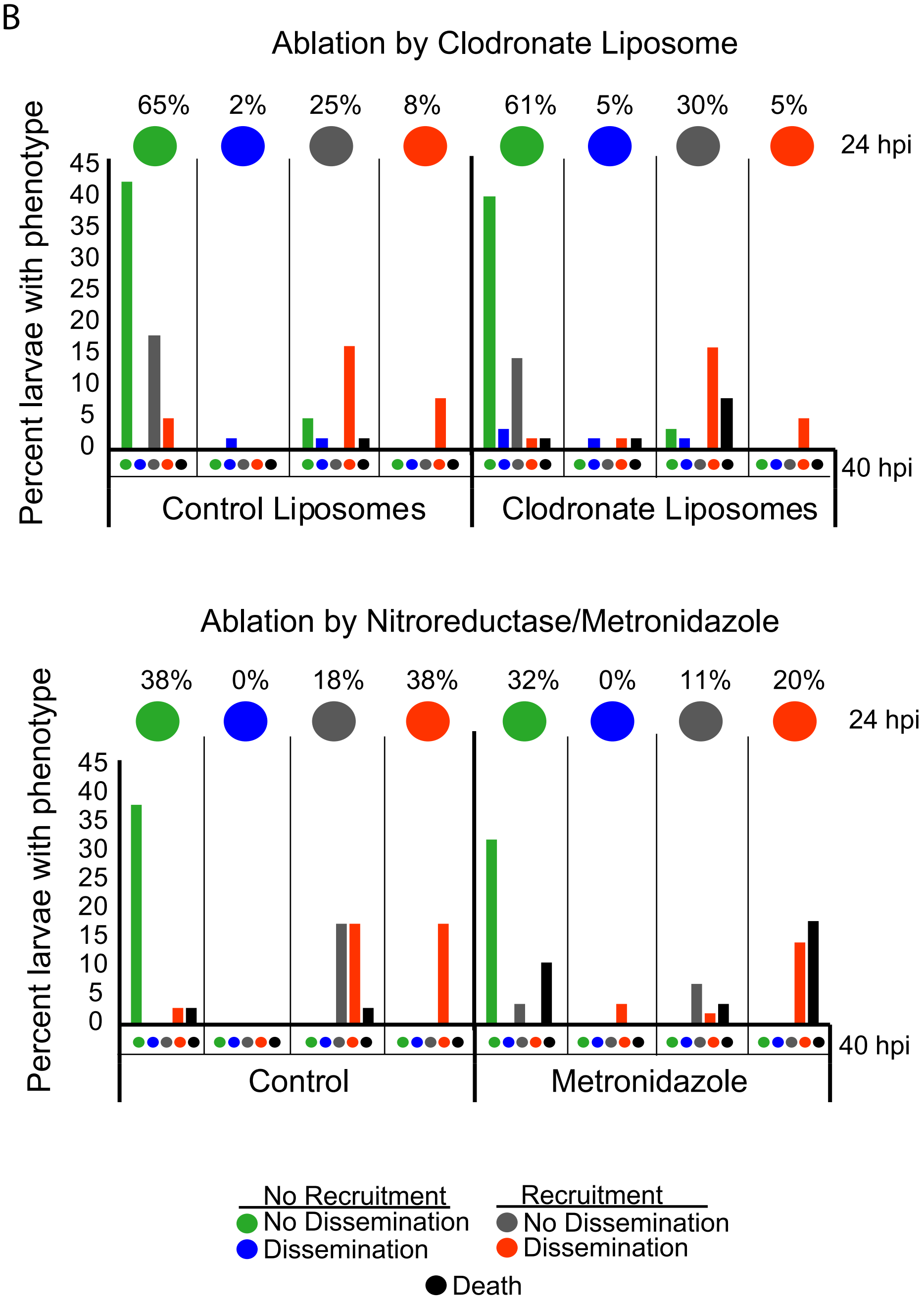

Fig. S7
