# Supplementary material for: Redundant Trojan horse and endothelial-circulatory mechanisms for host-mediated spread of Candida albicans yeast
Source: PLoS Pathog. 2020 Aug 10;16(8):e1008414. doi: 10.1371/journal.ppat.1008414 (PMC7447064; doi:10.1371/journal.ppat.1008414)
Supplement: S12 Fig — This document outlines the steps taken in the MATLAB workflows used for image quantification, referencing the MATLAB scripts that are included as Supporting Information. (PDF) [file ppat.1008414.s012.pdf]

<u>Workflow 1:</u>

Script 1: Allison_candida_amounts_used:

This script is run for each individual fish, and is used to find the amount of *Candida* in the yolk, the amount of disseminated *Candida*, and the distance from the yolk of the disseminated *Candida*. It reads in masks made in ImageJ of the *Candida*, yolk, and body of the fish and will export data into 3 Excel files as well as saving as 3 MATLAB files for use in other scripts (the other scripts combine data from each fish into one file).

Step-by-Step Description:

1. **Input labels** that you want the files to be saved as (examples below) and the number of that fish in its group.
    a. It is important to keep the names consistent and not to skip a number so that the next script can read the same file name for that group and go through each fish in the group by number
    b. X is the label for the data for the amount of *Candida* in the yolk
    c. Y is the label for the data for the amount of disseminated *Candida*
    d. Z is the label for the data for the distances of each *Candida* pixel
    e.
```
n = 30; %Number for this fish
  z= ['DMSO_',num2str(n)]; %Label for fish for distance
  x= ['DMSO_ca_yo_',num2str(n)]; % label for fish for candida in yolk
  y= ['DMSO_ca_diss_', num2str(n)]; % label for fish for disseminated candida
```
2. Read in the *Candida*, yolk, and fish body mask for that fish
    a. We had a separate folder for each fish with all the masks in it for that fish, so make sure you are in the appropriate folder. This will also allow that data to be saved in that folder for each fish.
3. Convert masks to MATLAB masks
4. Measure the amount of *Candida* present in the yolk
5. Remove *Candida* in yolk and save the rest of the *Candida* in a new matrix.
6. Remove any fluorescent "*Candida*" pixels outside of the fish
7. Measure amount of disseminated *Candida*
8. Make a distance map from the yolk mask
    a. This will measure the distance away from the yolk

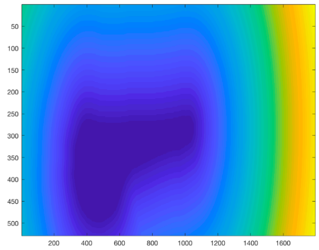

9. Measure the distance from the yolk of each pixel of *Candida*
10. Save data
    a. 3 Excel files and 3 MATLAB files
    b. MATLAB files with labels given for X & Y in step 1 will be used in the script: Allison_combining_candida_amounts_used (Script 2)
    c. MATLAB file with label Z (step 1) will be used in the script: New_real_Bins_used (Script 3)

Script 2: Allison_combining_candida_amounts_used
This script uses the MATLAB files that were saved for each fish for the *Candida* amounts (output x & y of Script 1: Allison_candida_amounts_used) and combines it into one sheet for amount of *Candida* in yolk and another sheet for the amount of disseminated *Candida*. It also sorts the fish further into different columns according to their respective groups.

Step-by-Step Description:

1. **Input** the total number of DMSO and TERF fish

   ```
   x=  44; %number of DMSO fish
   y=  32; %number of TERF fish
   ```

2. Read in MATLAB files for each fish putting into 4 sheets
   a. Amount of *Candida* in yolk for DMSO fish
   b. Amount of *Candida* in yolk for TERF fish
   c. Amount of disseminated *Candida* for DMSO fish
   d. Amount of disseminated *Candida* for TERF fish
3. Get Data values for each fish into the corresponding sheet
4. Put DMSO and TERF together into one sheet
5. Split fish into sheets for their group (8 Groups total)
   a. RAC & WT
   b. DMSO & TERF
   c. Dissemination & No Dissemination
6. Combine sheets back together so that there is one sheet with all 8 groups for amount of *Candida* in yolk and one for amount of disseminated *Candida*
7. Export data to Excel sheets

Script 3: New_real_Bins_used
This script uses the MATLAB files (z) exported from Script 1: Allison_candida_amounts_used containing the distance from the yolk for each *Candida* pixel. This script takes the data from each fish sorts them into their correct groups, bins the data into one-pixel width bins, and exports it as one sheet.

Step-by-Step Description:

1. **Input** number of DMSO and TERF fish
2. Find the max distance for each fish and put in its own matrix
3. Find the max distance value for all DMSO and all TERF fish
4. Round the max value up for DMSO and TERF
5. Find the max between DMSO and TERF
6. Bin numbers for each fish into the same number of bins as the max distance value for that fish
7. Sort fish into matrices for each group (8 groups total)
    a. DMSO & TERF
    b. RAC & WT
    c. Disseminated & Non-Disseminated
8. Find the number of fish in each group
9. Divide the number of pixels in each bin by the number of fish in that group to normalize
10. Sort back into one matrix with all 8 groups adding together the number of pixels in each bin for each fish
11. Make a matrix for fish with dissemination and fish without dissemination
12. Make new matrices with the same data but starting at a distance of 5 pixels away
13. Transform all matrices
14. Plot the data
15. Export data to Excel

<u>Workflow 2:</u>

Script 4: Candida_yolk_fli1_dist

This script uses masks made in ImageJ of *Candida*, the yolk, *fli1:EGFP*, and finds the amount of *Candida* in each fish, the distance of the *Candida* in the yolk to the edge of the yolk, and the distance of each pixel of *Candida* to *fli1:EGFP*. This script was run for each individual fish and the numbers recorded by hand.

Step-by-Step Description:

1. Read in masks for *Candida*, yolk, and *fli1:EGFP*
2. Convert masks to MATLAB masks
3. Remove *Candida* outside of the yolk
4. Make a distance map from the yolk mask
    a. Distance from inside of the yolk to outside of the yolk

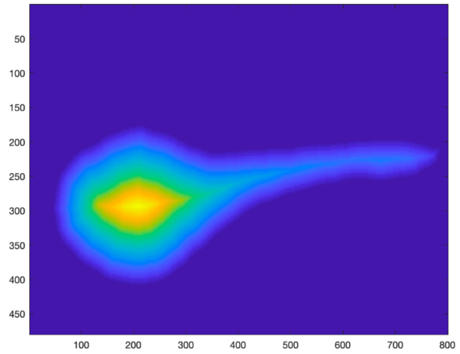

5. Make a distance map from *fli1:EGFP* mask
    a. Distance to *fli1:EGFP*+

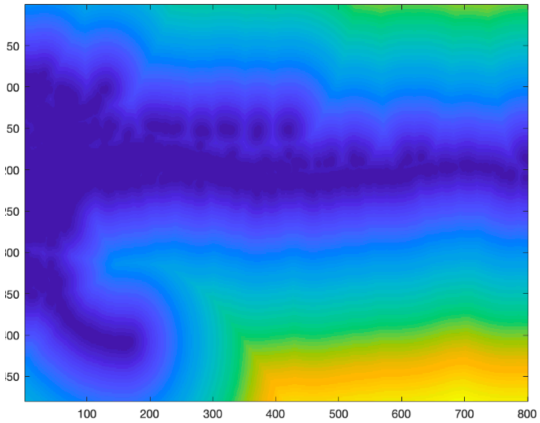

6. Find distance of each *Candida* pixel to edge of yolk
7. Find distance of each *Candida* pixel to *fli1:EGFP*
8. Find the mean distances to edge of yolk and to *fli1:EGFP*
9. Measure total amount of *Candida* in yolk

Workflow 3:

Script 5: New_TNF_express

This script uses masks made in ImageJ of the *Candida*, neutrophils, and TNFalpha and finds the amounts of TNFa, neutrophils, and TNF+ neutrophils at the infection site.

Step-by-Step Description:

1. Read in masks for *Candida*, neutrophils, and TNFa
2. Convert to MATLAB masks
3. Measure total *Candida* area
4. Measure amount of tnf area in *Candida* area
    a. Also TNF area as percent of *Candida* area
5. Measure amount of neutrophil area in *Candida* area
    a. Also neutrophil area as a percent of *Candida* are
6. Measure amount of TNF+ Neutrophil area in *Candida* are
    a. Also as a percent of *Candida* area
7. Determine % of neutrophils at infection sites expressing TNF
8. Export to Excel
